# A paper-based dual functional biosensor for safe and user-friendly point-of-care urine analysis†

**DOI:** 10.1039/d4lc00163j

**Published:** 2024-04-12

**Authors:** Yujia Li, Yingqi Kong, Yubing Hu, Yixuan Li, Rica Asrosa, Wenyu Zhang, Buddha Deka Boruah, Ali K. Yetisen, Andrew Davenport, Tung-Chun Lee, Bing Li

**Affiliations:** a Institute for Materials Discovery, University College London London WC1E 7JE UK bing.li@ucl.ac.uk; b Department of Chemistry, University College London London WC1E 7JE UK; c Department of Chemical Engineering, Imperial College London London SW7 2AZ UK; d Department of Physics, Faculty of Mathematics and Natural Science, Universitas Sumatera Utara Medan 20155 Sumatera Utara Indonesia; e Department of Materials, University of Oxford Parks Road Oxford OX1 3PH UK; f UCL Department of Renal Medicine, Royal Free Hospital, University College London Rowland Hill Street London NW3 2PF UK

## Abstract

Safe, accurate, and reliable analysis of urinary biomarkers is clinically important for early detection and monitoring of the progression of chronic kidney disease (CKD), as it has become one of the world's most prevalent non-communicable diseases. However, current technologies for measuring urinary biomarkers are either time-consuming and limited to well-equipped hospitals or lack the necessary sensitivity for quantitative analysis and post a health risk to frontline practitioners. Here we report a robust paper-based dual functional biosensor, which is integrated with the clinical urine sampling vial, for the simultaneous and quantitative analysis of pH and glucose in urine. The pH sensor was fabricated by electrochemically depositing IrOx onto a paper substrate using optimised parameters, which enabled an ultrahigh sensitivity of 71.58 mV pH^−1^. Glucose oxidase (GOx) was used in combination with an electrochemically deposited Prussian blue layer for the detection of glucose, and its performance was enhanced by gold nanoparticles (AuNPs), chitosan, and graphite composites, achieving a sensitivity of 1.5 μA mM^−1^. This dual function biosensor was validated using clinical urine samples, where a correlation coefficient of 0.96 for pH and 0.98 for glucose detection was achieved with commercial methods as references. More importantly, the urine sampling vial was kept sealed throughout the sample-to-result process, which minimised the health risk to frontline practitioners and simplified the diagnostic procedures. This diagnostic platform, therefore, holds high promise as a rapid, accurate, safe, and user-friendly point-of-care (POC) technology for the analysis of urinary biomarkers in frontline clinical settings.

## Abstract 2

A safe, accurate, and reliable sensing platform for urinary biomarkers analysis of urinary biomarkers is clinically important for the early detection and monitoring of the progression of chronic kidney disease (CKD), as it has become one of the world's most prevalent non-communicable diseases.

## Graphical abstract

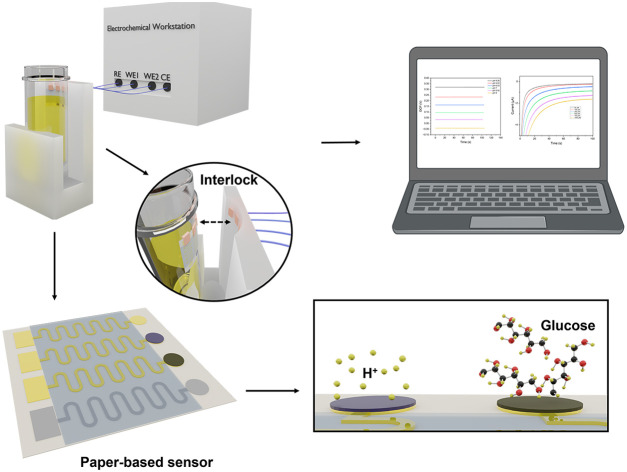

## 1. Introduction

CKD is the most serious non-communicable disease affecting the adult population worldwide. During its early stages, most patients remain asymptomatic, until they reach the end stage when dialysis treatments become necessary.^1^ Recently studies have reported the diagnosis of CKD using relatively easily measurable urinary biomarkers such as pH and glucose^2,3^ instead of the conventional analysis of glomerular filtration rate or blood albumin concentration.^4,5^ However, their accurate analysis relies on sending the samples to laboratories, where dedicated equipment and skilled personnel are required. Although technically both pH and glucose could be measured in frontline clinical settings using urine test strips or dipsticks,^6,7^ in the absence of a quantitative readout device, these methods provide only an approximate range instead of an accurate value and are susceptible to interference, which makes accurate biomarker concentration-based disease diagnosis and tracking a challenge. Additionally, the handling or dipstick testing of these body fluid samples, which may contain various types of pathogens, requires multiple steps of pretreatment and sample transfer. This leads to an increased health risk from the accidental splashing of and exposure to the body fluids from patients, which account for 15–60% of the total accidents in different clinical settings.^8,9^ As such, the development of an accurate, safe, and user-friendly point-of-care platform for this urinary analysis is clinically important and urgent for both patients and practitioners.

For urinary pH, a lower fasting value (5.0–5.5 in CKD samples compared with 6.5–7.0 in healthy controls) presents a higher CKD risk.^2^ Currently, optical sensors have been developed to measure the urine pH within this range using different reading strategies, such as naked-eye colorimetric^10^ and fluorometric methods.^11^ However, like the dipstick method, it is difficult to accurately determine the colour changes when environmental light changes or the pH variation is small. Although these methods can be improved by integrating a digital camera for more accurate readings,^12^ objective factors, such as illumination intensity, imaging distance and angle, and different calibration algorithms, made it challenging to obtain accurate results.^13,14^ On the other hand, a wide range of pH sensitive materials and structures have been incorporated into working electrodes for the development of electrochemical pH sensors, which have demonstrated a higher accuracy and ease to be integrated into various biomedical applications compared to optical sensors.^15^ For example, Mazzaracchio *et al.* developed an iridium oxide-based pH sensor, which presented a super-Nernst response of 79 ± 2 mV per pH, with a negligible interference effect from common sweat ions, including Na^+^, K^+^, and Cl^−^.^16^ The IrO_2_ based pH sensors hold several advantages, such as good stability over a large range of pH, temperatures, and pressures, good durability, and particularly good biocompatibility, which make IrO_2_ one of the most popular materials for pH sensing applications.

On the other hand, the presence of urinary glucose has been found with the increased risk of mid-to-advanced stage CKD.^17^ Current technologies for the determination of urinary glucose can also be divided into optical and electrochemical methods. The optical glucose sensors mostly rely on a combination of enzyme and redox indicators, where the enzyme firstly reacts with glucose to produce H_2_O_2_, which then reacts with the redox indicators to produce a colour change or fluorescence signal for a quantitative analysis of glucose.^18,19^ The glucose concentration can also be quantitatively analysed by detecting the oxygen changes during the redox process.^20^ However, like the optical pH sensors, it is a challenge for optical glucose sensors to provide accurate results under varying environmental and sensing conditions. As for the electrochemical glucose sensors, although four generations of sensing strategies have been reported,^21^ the first one, which relied on the enzymic oxidation of glucose and successive oxidation of H_2_O_2_,^22^ remains the most attractive method in both scientific and industrial settings due to its simple design, high sensitivity, selectivity of enzymes, and good biocompatibility.^23–25^ However, in this configuration, when the GOx are in direct contact with the electrode or interference proteins deposit around the redox centre of GOx, its enzymatic activity and the electron transport efficiency between GOx and the electrode will be significantly reduced.^26,27^ To tackle this issue, many methods have been demonstrated to be effective, such as using chitosan as an isolation and protection layer for GOx^28^ and nanomaterials to shorten the electron transport distance between the redox centre and the electrode surface.^29,30^ Overall, electrochemical glucose sensors offer several distinct advantages over optical sensors, including high sensitivity and accuracy, being inert to the variation of sample colour or turbidity, wider dynamic detection range, faster response times, and ease of integration into different biomedical applications.^21^

In this work, we developed a disposable paper-based dual functional integrated sensing platform for the simultaneous analysis of urinary pH and glucose. This platform is compatible with the current large-scale device production techniques, which make it cost-effective (with an expected cost of ∼£8.24 for 100 sensors and a reusable digital readout platform of ∼£20, as demonstrated in Table S1†). Additionally, this platform has been creatively integrated into the UK National Health Service (NHS) urine sampling vial. Users can easily start the test by simply filling the sampling vial with urine and plugging the vial into the POC readout for analysis. The IrOx layer of the pH sensor was electrochemically deposited using an optimised constant-voltage method, which enabled a super Nernst sensitivity and a long-term stability. The AuNPs, together with a graphite framework, were used as signal amplifiers to enhance the detection of glucose using a GOx and Prussian blue pair, by providing a higher surface area and an increased electrochemical activity on the electrode surface.^31^ Additionally, the entire analysis is a plug-in process, which does not require sample transfer or opening of the urine sampling vial, which largely reduces the health risk to the frontline practitioners and simplified the diagnostic procedures. We validated this robust and accurate sensing platform in artificial urine and performed the POC detection in clinical samples, where a superior limit of detection (LOD), sensitivity, and accuracy over a wide detection range were observed together with good stability. This paves the way for a new strategy for the accurate, safe, and user-friendly POC monitoring of CKD in primary care settings, such as GP surgeries or even patients' homes.

## 2. Experimental section

### 2.1. Materials and reagents

Paper substrates, both sides of which were coated with silicone rubber, and commercial enzyme colorimetric glucose detection kits (referred to as dipstick methods) were purchased from commercial suppliers. The SU-8 negative photoresist and the corresponding developer were from A-Gas. The chemicals used in this project include sulfuric acid (H_2_SO_4_), tetrachloroauric(iii) acid (HAuCl_4_), potassium hexacyanoferrate(iii) (K_3_Fe(CN)_6_), hydrogen chloride (HCl, 37%), chitosan, graphite, glucose oxidase (GOx), bovine serum albumin (BSA), glutaraldehyde (25%), Nafion 117 (5%), iridium tetrachloride (IrCl_4_), hydrogen peroxide (H_2_O_2_, 30%), sodium sulfate (Na_2_SO_4_), calcium chloride (CaCl_2_), potassium oxalate (K_2_C_2_O_4_), sodium phosphate monobasic dihydrate (NaH_2_PO_4_·2H_2_O), sodium phosphate dibasic dihydrate (Na_2_HPO_4_·2H_2_O), sodium citrate tribasic dihydrate (HOC(COONa)(CH_2_COONa)_2_·2H_2_O), boric acid (H_3_BO_3_), phosphoric acid (H_3_PO_4_), acetic acid (CH_3_COOH), human serum albumin, glycine, uric acid, sarcosine, urea, l-ascorbic acid, creatinine, and potassium hexacyanoferrate(ii) trihydrate (K_4_Fe(CN)_6_·3H_2_O), which were purchased from Sigma-Aldrich. Potassium chloride (KCl) was purchased from Alfa Aesar. Iron(iii) chloride (FeCl_3_) and oxalic acid (C_2_H_2_O_4_) were purchased from Acros Organics. Phosphate-buffered saline (PBS) was purchased from Fisher Bioreagents. Potassium carbonate (K_2_CO_3_) and sodium hydroxide (NaOH) were purchased from Fisher Scientific. d-Glucose was purchased from Gibco. Sodium chloride (NaCl), ammonium chloride (NH_4_Cl), and sodium hypochlorite (NaClO) were purchased from VWR. Magnesium sulfate (MgSO_4_) was purchased from Thermo Fisher. All chemicals, unless specified, were of analytical reagent grade.

### 2.2. Fabrication and characterisation of the paper substrate and base electrodes

Silicone rubber coated paper was chosen as the substrate (weight of 37.83 g m^−2^, thickness of 5 × 10^−2^ mm), on which we firstly sputtered (Q150V Plus, Quorum) 15 nm Cr as an adhesive layer followed by 150 nm gold as the working and counter electrodes,^32^ and 150 nm silver as the reference electrode. SU-8 photoresist, used as the electrode encapsulation material, was spin-coated onto the paper-based electrodes at 3000 rpm for 20 s and then placed on a hotplate at 95 °C for 30 min. The electrodes were cooled to room temperature after this soft baking, then exposed to 365 nm UV light (Honle UV Technology) for curing. Then, the samples were developed in propylene glycol methyl ether acetate (PGMEA), rinsed with IPA and DI water, and dried with a nitrogen gun. A 14% NaClO aqueous solution was dropped onto the silver reference electrode to react for 1 min until the silver turned dark and then was rinsed with DI water and blown dry with a nitrogen gun.

The surface morphology of the paper substrate and paper-based flexible electrodes was characterised using scanning electron microscopy (SEM, Hitachi and Zeiss) and a stylus profilometer (Bruker). The elemental mapping of the resulting Ag/AgCl reference electrode was achieved using an energy-dispersive spectrometer (EDS, Zeiss). The electrical resistance of the gold electrodes was measured using a semiconductor analyser (Keysight).

### 2.3. Fabrication and characterisation of the pH sensor

The electrochemical deposition of IrOx was achieved as follows. Firstly 0.15 g IrCl_4_ was dissolved in 100 mL DI water with magnetic stirring for 3 min. Then H_2_O_2_ (1 mL, 30 wt%) and (COOH)_2_·2H_2_O (0.5 g) were consecutively added into the above solution and both followed by a 10 min stirring. K_2_CO_3_ was used to adjust the solution pH to 10.5 and this resulting solution was kept in the dark for 48 h to stabilize, indicated by a colour change from yellow to light purple. The solution was stored in the dark at 4 °C when not in use. The chronoamperometric method with a voltage of 0.7 V was applied to the Au working electrode for 30 min, which resulted in a dark blue IrOx film on the electrode surface (Fig. S1†). Britton–Robinson (BR) buffers, which contained boric acid (0.04 M), phosphoric acid (0.04 M), and acetic acid (0.04 M), were prepared and adjusted with NaOH (0.2 M) to obtain solutions with different pH values.^33^

Open circuit potential (OCP) measurements on these pH sensors were performed using an electrochemical workstation (Metrohm PGSTAT204) in buffer solutions with different pH values for 100 seconds under stable conditions. The IrOx modified electrode was used as the working electrode and the Ag/AgCl electrode as the reference electrode. The selective coefficients of four interferents (0.1 mM NaCl, 0.1 mM KCl, 0.1 mM NH_4_Cl and 0.1 mM CaCl_2_) were studied using potentiometric method.

The sensitivity of the pH sensor can be calculated by the following equation,1
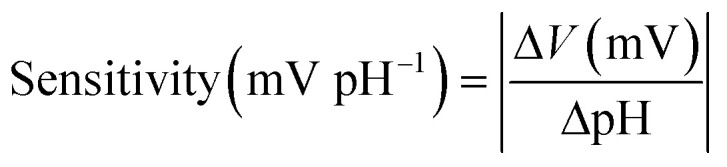
where Δ*V* is the change of the OCP potentials and ΔpH is the change of pH.

### 2.4. Fabrication and characterisation of the glucose sensor

The working principle of the enzyme-based electrochemical glucose sensor was based on the following reaction:
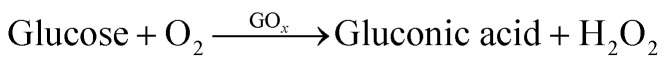
H_2_O_2_ → 2H^+^ + O_2_ + 2e^−^This involves the oxidation of glucose to form gluconic acid and H_2_O_2_, and the resulting H_2_O_2_ is then oxidised at the electrode surface, generating electrons proportional to the glucose concentration.

The Au working electrode was firstly cleaned by cyclic voltammetry (CV) in H_2_SO_4_ (50 mM) solution for 10 cycles, with a potential from −0.4 to 1 V and a scan rate of 100 mV s^−1^. Then the electrode was immersed into HAuCl_4_ (2 mM) solution with H_2_SO_4_ (2 M) as the solvent with a chronoamperometric potential of −0.1 V on the surface for 30 min for AuNP deposition, where an Au counter electrode and an Ag/AgCl reference electrode were used in a three-electrode system (Fig. S2†). Then Prussian blue, as a H_2_O_2_ sensitive layer, was electrochemically deposited by immersing the working electrode into a 50 mL Prussian blue solution (100 mM KCl, 2.5 mM FeCl_3_ and 2.5 mM K_3_Fe(CN)_6_ in 100 mM HCl) and running the CV from 0 to 0.5 V at a scan rate of 20 mV s^−1^ for 4 cycles (Fig. S3†). The GOx was immobilised on top of the Prussian blue layer *via* a chitosan/graphite framework. Chitosan was firstly dissolved in 2% acetic acid with magnetic stirring for 1 h at 70 °C to prepare a 1.0 wt% chitosan solution. Exfoliated graphite at 5 mg mL^−1^ was added and ultrasonicated for 1.5 h. GOx and BSA were dissolved in 0.01 M PBS solution (pH 7.4) at concentrations of 50 mg mL^−1^ and 10 mg mL^−1^, respectively. Then the chitosan/graphite solution was added and mixed into this GOx solution at a volume ratio of 2 : 1. 0.5 μL of the GOx/BSA/chitosan/graphite composite was drop cast on the Prussian blue coated electrode and dried at room temperature for 30 min. 0.5 μL glutaraldehyde solution (2 wt%), used as the material to keep the enzyme active, was coated onto this electrode and kept in the refrigerator at 4 °C to dry. Lastly, Nafion (0.8 μL, 0.5 wt%), as the enzyme protective layer, was dropped onto the electrode and dried at room temperature. Samples were stored at 4 °C before and after use.

PBS buffer with different glucose concentrations was prepared by adding appropriate amounts of glucose to PBS buffer and stored at 4 °C when not in use. Artificial urine was prepared according to a previous study by Sarigul *et al.*^34^ It contained Na_2_SO_4_ (11.97 mM), uric acid (1.49 mM), HOC(COONa)(CH_2_COONa)_2_·2H_2_O (2.45 mM), creatinine (7.79 mM), urea (249.75 mM), KCl (30.95 mM), NaCl (30.05 mM), CaCl_2_ (1.66 mM), NH_4_Cl (23.67 mM), K_2_C_2_O_4_ (0.19 mM), MgSO_4_ (4.39 mM), (NaH_2_PO_4_·2H_2_O (18.67 mM), and Na_2_HPO_4_·2H_2_O (4.67 mM). Glucose was added to the artificial urine to provide samples with different glucose concentrations.

Surface morphology was characterised using SEM. The electrochemically active surface area (ECSA) of the modified electrodes was measured by CV in the electrolyte solution containing K_3_Fe(CN)_6_ (10 mM), K_4_Fe(CN)_6_ (10 mM), and KCl (1.0 M), shown in Fig. S4.† The immobilisation of GOx was confirmed using Fourier-transform infrared (FTIR) spectroscopy (PerkinElmer, UK). The glucose sensor was characterised by chronoamperometry in PBS solution and artificial urine, which were spiked with glucose at different concentrations. Steady-state currents were obtained after 10 min. Glucose measurements were achieved using chronoamperometry (−0.1 V for 100 s) after 10 min, when the steady state was reached, then at 5 min intervals to ensure homogeneity of the solution. The sensitivity of the glucose sensor can be calculated by either of the following equations,^35^2
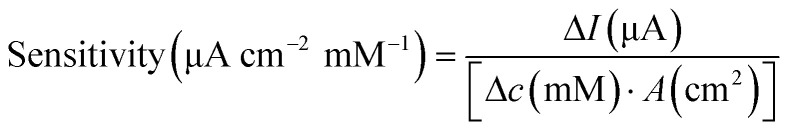
3
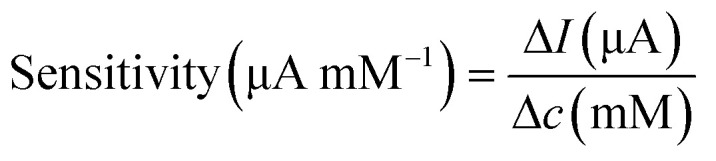
where Δ*I* is the change of the current, Δ*c* is the change of the glucose concentration in mM, and *A* is the area of the electrode. The interference test was performed by adding the following materials into 0.1 M PBS solution (pH 7.4) to reach a specified concentration: glucose (1000 μM), uric acid (50 μM), ascorbic acid (10 μM), BSA (50 μM), human serum albumin (10 μM), glycine (50 μM), creatinine (50 μM), sarcosine (50 μM), and urea (50 μM). The LOD of both the pH and glucose sensor was determined by the following equation,^36^

4 LOD = 3.3*S*_b_/*S*

where *S*_b_ is the standard deviation of the noise and *S* is the slope of the linear calibration curve.

## 3. Results and discussion

### 3.1. Overview of dual functional sensing platform

**Fig. 1:**
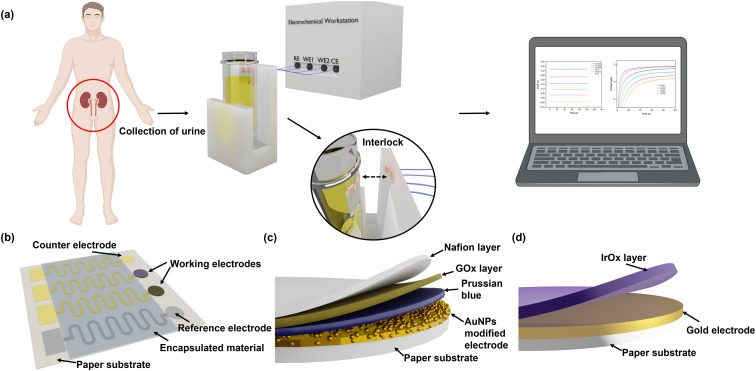
Schematic of the POC dual functional sensor for urine pH and glucose analysis. (a) Workflow and prototype device integrated with NHS urine sampling vial for POC urine pH and glucose analysis. (b) Paper-based substrate with Au as the working and the counter electrodes and Ag/AgCl as the reference electrode. Serpentine connectors enhance the flexibility of the electrodes and are encapsulated using an SU8 layer. (c) Structure of glucose sensor. GOx and Prussian blue are used as the sensing layer, while AuNPs and graphite composite are used as the signal amplifier. (d) Structure of IrOx-based pH sensor.

The workflow and schematic of this paper-based dual functional sensing platform for the POC analysis of human urine is shown in Fig. 1. The paper-based sensor has been adhered to the inner wall of a urine sampling vial, which has been widely used in UK hospitals. Once the urine sample has been loaded, the vial can be inserted into a vial holder, which has surface connection pins to the sensor electrodes and is interfaced with a portable potentiostat. The results can be remotely sent to GPs or other healthcare professionals for further assessment, as shown in Fig. 1(a). Fig. 1(b) presents the design of this paper-based flexible sensor, which uses cellulose fibres as the main substrate component. Since cellulose fibres present weak adhesion to metal electrode materials,^37^ silicone rubber was chosen as an intermediary layer for contact electrodes to sit on and the SU-8 photoresist was used as an encapsulation layer to prevent any crosstalk between different electrodes in ion-rich solutions. Then custom-shaped metal electrodes, with 15 nm Cr as the adhesive layer and 150 nm Au or Ag as the electrode materials, are sputtered onto the silicone rubber through a laser-cut hard mask. In this configuration, each dual functional sensor consists of two differently functionalised working electrodes for pH and glucose sensing, one Ag/AgCl reference electrode, and one Au counter electrode. All electrodes had the serpentine design to effectively increase their durability and reduce stress when attached to the curved structures.^38^Fig. 1(c) shows the 3D schematic of the glucose sensor, where the Au electrode was first decorated with AuNPs to increase the surface area. Then, Prussian blue and GOx were used as a pair for the oxidation of glucose and H_2_O_2_, respectively, to achieve indirect glucose sensing. To enhance the electron transport rate and amplify the sensing signals, GOx was loaded into a chitosan and graphite framework and then protected by a Nafion layer. Lastly, as shown in Fig. 1(d), we electrochemically deposited a layer of IrOx under optimal conditions onto the Au working electrode for a robust pH sensing layer. Commercial silicone rubber coated paper was chosen as the substrate mainly because of its suitable surface 3D structure for further modification, cost-effectiveness, environmental friendliness, and potential to be produced at large scales. The silicone rubber sandwiched paper uses fewer synthetic materials, which makes it more sustainable and biodegradable, while the production method is compatible with current industrial methods, which makes it promising for large-scale production and deployment, especially in resource-limited settings.

### 3.2. Characterisation of paper-based flexible substrate and electrodes

**Fig. 2:**
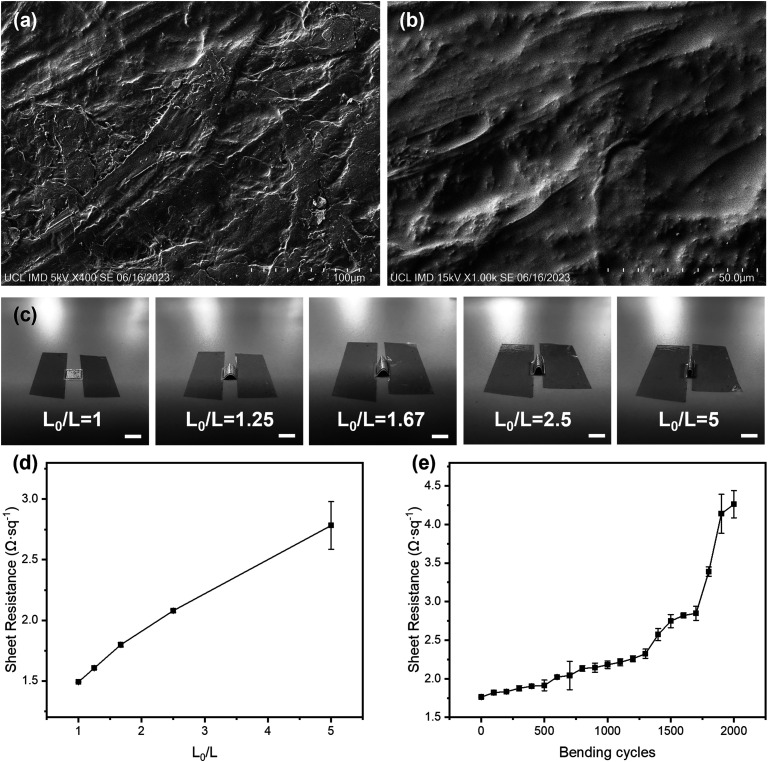
Characterisation of paper-based substrate and flexible electrodes. SEM images of (a) silicone rubber coated paper substrate and (b) gold electrodes on the paper-based substrate. (c) Five different bending states defined by the ratio of arc length (*L*_0_) and chord length (*L*) (scale bar: 5 mm). (d) Sheet resistance of the paper-based electrode changed at different bending degrees (error bars are based on 3 measurements of the same electrode). (e) Sheet resistance changes of the paper-based electrode at 1.67 bending degree with 0–2000 bending cycles (error bars are based on 3 measurements of the same electrode).

The flexibility and integrity of the substrate and the electrodes greatly affect the sensing performance of the electrochemical sensing platform. SEM and 3D mapping have been used to characterise their surface morphologies. From Fig. 2(a) and (b) and S5,† it can be seen that the original surface morphology of the cellulose fibre has been preserved, which facilitates the adhesion of the Cr/Au electrodes and the electrodeposited AuNPs and IrOx layers.^39^ Although the Cr/Au electrodes are coated onto this 3D substrate, due to the heterogeneous deposition directions, their structural integrity remained excellent and their sheet resistance was determined to be around 1.49 Ω sq^−1^, which is ideal for the rapid charge transfer and exchange during the redox reactions of glucose detection and pH sensing. In addition, benefiting from the flexible cellulose paper substrate, the Cr/Au electrodes maintain excellent electrical performance when being repeatedly bent. As shown in Fig. 2(c) and (d), the paper-based electrodes have been bent to different degrees, which are defined using the ratio of arc length (*L*_0_) and chord length (*L*) (*L*_0_/*L*) from 1 to 5 (from flat to 180° of bending). Meanwhile the sheet resistance showed only a minor change from 1.49 to 2.78 Ω sq^−1^, which remained low (4.26 Ω sq^−1^) even after 2000 cycles at *L*_0_/*L* = 1.67, indicating its excellent stability and flexibility, as shown in Fig. 2(e). This paper-based platform enables the production of the sensor at low cost, which is suitable for large-scale diagnostic and screening purposes.

Commercial Ag/AgCl reference electrodes are usually bulky and not feasible to be integrated with planar sensing platforms.^40^ In this study, a miniaturised pseudo-Ag/AgCl reference electrode was fabricated by post processing of sputtered Ag electrodes with NaClO. As shown in the SEM image (Fig. S6a†), AgCl particles are uniformly and firmly distributed across the reference electrode surface. The average particle size of AgCl on the electrode surface was 0.86 μm (Fig. S6b†). The ratio of Ag and Cl elements was determined to be 1.3 : 1 using EDS (Fig. S7†), indicating the Ag/AgCl mixture composition when AgCl is formed during the post-processing. We cross-checked the OCP performance of our Ag/AgCl reference electrode against a commercial Ag/AgCl reference electrode in 3.5 M KCl solution, where the former was used as a working electrode and the latter as a reference electrode (Fig. S8†). The potential difference between these two electrodes was 7.03 mV at the beginning with the average remaining at around 6.58 mV over 10 min, which demonstrated the excellent stability of our pseudo reference electrode. The performance of this pseudo reference electrode was further compared with that of a commercial one using CV in the [Fe(CN)_6_]^3−/4−^ system, where shifts of 52 mV and 49 mV were observed for the anodic (oxidation) and cathodic (reduction) peaks, respectively (Fig. S9†). This indicated the good performance of the pseudo reference electrode, in agreement with a previous work from Rohaizad.^41^

### 3.3. pH sensors and characterisation

**Fig. 3:**
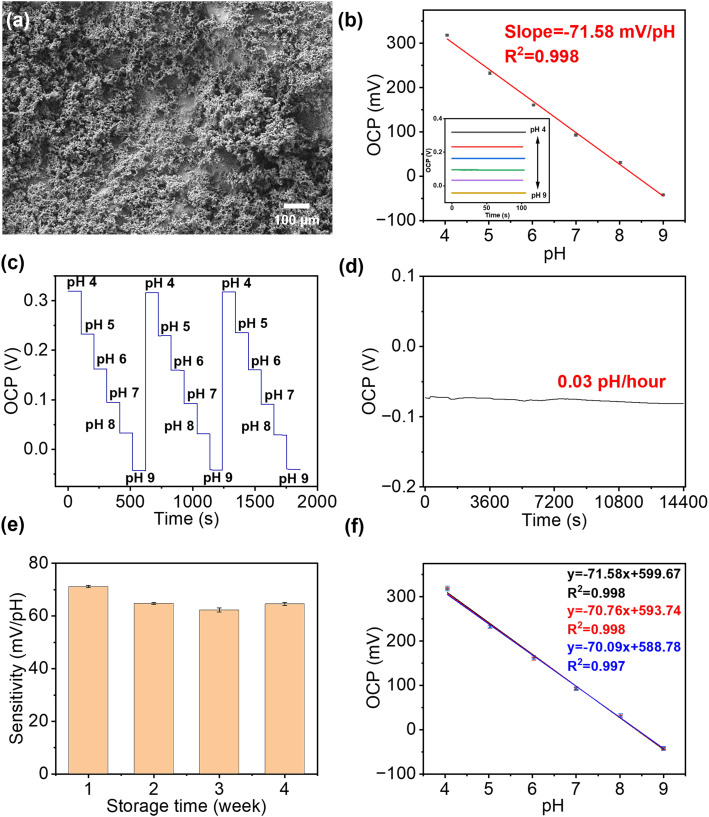
Electrochemical sensing performance of the paper-based pH sensor. (a) SEM image of the IrOx film on paper-based gold electrodes. (b) The calibration plot of OCP at different pH values in BR buffer (*n* = 3). The inset shows the OCP responses of the pH sensor for increasing pH from 4 to 9. (c) Reusability of pH sensor in buffers with different pH values. (d) Continuously monitoring of OCP value in buffer solution at pH 9 for 4 h showed a drop rate at 0.03 pH h^−1^ (frequency: 0.1 s). (e) The long-term stability of this pH sensor over four weeks. (f) Plot of OCP measured using three independently prepared sensors in buffers, which showed good reproducibility over a pH range from 4 to 9.

The SEM image in Fig. 3(a) showed that IrOx had been successfully and uniformly deposited onto the surface of the Au working electrode with the appearance of porous clusters. Normally, the response of smooth anhydrous iridium oxide film to pH changes can be described as IrO_2_ + H^+^ + e^−^ ↔ IrO·OH,^42^ where the ratio of H^+^ to e^−^ is 1 : 1; thus it follows a Nernst response of 59 mV pH^−1^. However, in this work, the hydrated IrOx was deposited onto the Au surface as porous clusters, which have various morphologies and compositions. This cluster layer response to pH changes follows a different equation below,^38^(1 − *x*)Ir(OH)_3_ + *x*Ir(OH)_2_O^−^ + *x*H^+^ ↔ (1 − *y*)IrO(OH)_2_ + *y*IrO(OH)O^−^ + (1 + *y*)H^+^ + e^−^where each H^+^ corresponds to (1 + *y* − *x*)^−1^ e^−^. This value varies between 1 (when only anhydrous iridium oxide is present) and 1.5 (when only hydrated iridium oxide is present), which led to a sensitivity between 59 mV pH^−1^ and the theoretical limit of 90 mV pH^−1^ with the most observed around 50–75 mV pH^−1^ at room temperature.

The performance of these pH sensors was firstly characterised in BR buffer solutions with different pH, in which the steady-state OCP values were recorded, as shown in Fig. 3(b). This calibration plot of OCP showed that the potential linearly decreased with increasing pH over the range of 4–9 (clinically rarely below 5 and above 9), with a sensitivity of 71.58 mV pH^−1^ in the BR buffer solution, with *R*^2^ = 0.998, and a LOD value of 0.07 pH can be obtained. This sensitivity is in agreement with previous reports on electrochemically deposited IrOx electrodes.^40,43^ Then, the cyclic measurements were carried out in buffer solutions at different pH values to characterise the reusability of these pH sensors, as shown in Fig. 3(c). The OCP of pH sensors remained constant after three cycles, demonstrating an excellent reusability. As shown in Fig. 3(d), the short-term stability of this pH sensor has also been tested by continuously monitoring the OCP in a buffer solution at a pH of 9 for 4 h, which showed only a small drift at 0.03 pH h^−1^, indicating good durability of the sensor. On the other hand, it is also crucial for a biosensor to remain accurate and consistent over the long-term. To test the shelf-life, the sensors were kept in sealed Petri dishes at 4 °C for a span of over four weeks. As shown in Fig. 3(e), the pH sensor maintained 87.5% of the maximum signal (initial response) at room temperature after 4 weeks storage with RSD values below 4.58%, which indicated good and an adequately long stability for most of biomedical applications.

The reproducibility of the fabrication method (batch-to-batch difference) has also been studied, as shown in Fig. 3(f), where three pH sensors produced using identical process show highly reproducible readings in the same buffer solution with pH values from 4 to 9.

The selectivity of the pH sensor is another important criterion to be characterised, as many ions and molecules existing in patient urine samples may interfere with H^+^ on the sensor surface. Therefore, the separate solution method (SSM) has been used to study the interference effect caused by the most frequently found ions in urine, including Na^+^, K^+^, NH_4_^+^, and Ca^2+^ ions.^34^ This effect is quantified by potentiometric selectivity coefficients described by the equation,^44^5
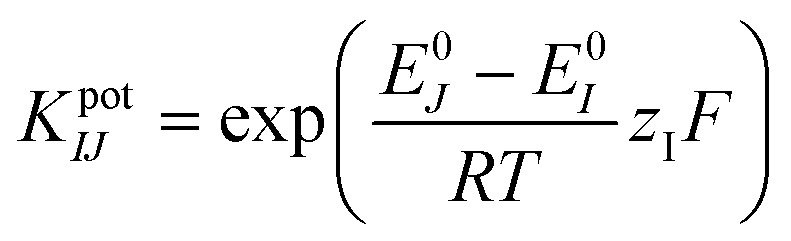
where *K*^pot^_*IJ*_ is the potentiometric selective coefficient, *E*^0^_*J*_ the sensing potential of the interfering ion, *E*^0^_*I*_ the sensing potential of the primary ion, *z*_*I*_ the charge numbers of the principal ion, *R* the gas constant, *T* the temperature, and *F* the Faraday constant. In this case, all ion selectivity coefficients have been determined to be below 10^−2^, which means that the sensor has a good ion selectivity in urine and is in agreement with previous literature (Table 1).^45–48^

**Table 1:** The selectivity coefficients of the pH sensor^a^

Ion (*J*)	*E* ^0^_*I*_ (V)	*E* ^0^_*J*_ (V)	*K* ^pot^_*IJ*_
K^+^	0.318	0.139	9.44 × 10^−4^
Na^+^	0.318	0.1604	2.17 × 10^−3^
NH_4_^+^	0.318	0.1038	2.40 × 10^−4^
Ca^2+^	0.318	0.1686	2.99 × 10^−3^

a The selectivity coefficients were obtained *via* the SSM method, and the concentrations of the primary ion needed to be equal to those of interfering ions. 0.1 mM H^+^ according to pH 4, which is the minimum pH value for the sensor's operating range.

### 3.4. Glucose sensors and characterisation

**Fig. 4:**
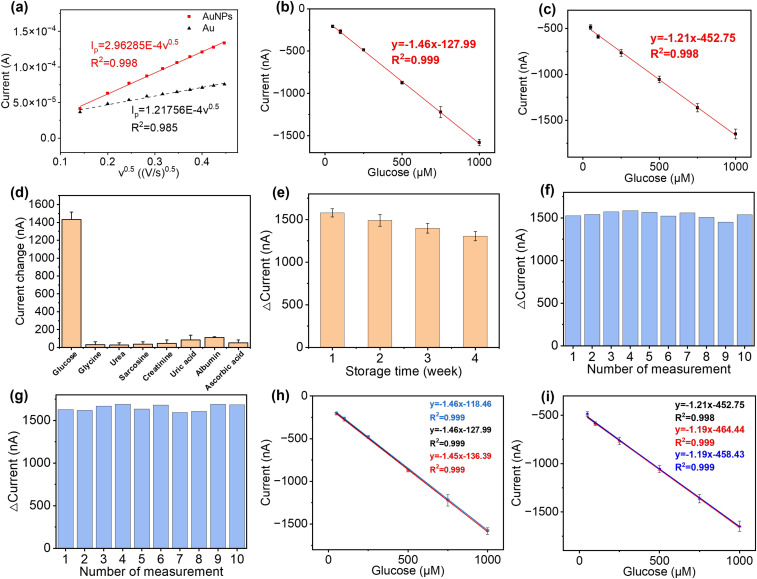
Performance test of paper-based glucose sensor. (a) Square root of the scan rate *vs.* peak current of the bare Au and AuNP electrode. (b) Detection of glucose in PBS solution with glucose concentration from 50 to 1000 μM (*n* = 3). (c) Detection of glucose in artificial urine with increasing concentration from 50 to 1000 μM (*n* = 3). (d) Specificity analysis against the common urine interferents in the presence of 1000 μM glucose, 100 μM glycine, 350 mM urea, 50 μM sarcosine, 10 mM creatinine, 5.5 mM uric acid, 300 mg L^−1^ albumin, and 0.56 mM ascorbic acid. (e) Long-term stability test for 4 weeks with measurement taken every week. Sensors were stored at 4 °C between measurements. Reusability analysis for the detection of 1000 μM glucose in (f) PBS buffer and (g) artificial urine. Reproducibility (batch-to-batch difference) of glucose detection ranging from 50 to 1000 μM in (h) PBS buffer and (i) artificial urine.

The AuNPs, together with a graphite framework, were used as the signal amplifiers to enhance the detection of glucose by providing a higher surface area and an increased electrochemical activity on the electrode surface. AuNPs were electrochemically deposited onto the surface of the bare Au electrode (Fig. S2b†). SEM images of electrochemically deposited Prussian blue (Fig. S10a), drop-casted enzyme film (Fig. S10b), drop-casted glutaraldehyde and Nafion film (Fig. S10c) are in the ESI.† The ECSA of the bare Au electrode and Au/AuNP electrode was measured using CV in K_3_Fe(CN)_6_ (10 mM) PBS solution and calculated following the Randles–Sevcik equation,

6 *I*_p_ = 2.69 × 10^5^*AD*^1/2^*n*^3/2^*v*^1/2^*C*

where *A* is the electrode-active area (cm^2^), *D* the diffusion coefficient (cm^2^ s^−1^), *n* the number of electrons transferred in the redox reaction, *C* the concentration of the reactant (mol cm^−3^), *I*_p_ the redox peak current (A), and *v* the scan rate of CV (V s^−1^). In this case, the ECSA of the Au electrode was determined to be 0.0707 cm^2^, and following decoration of AuNPs on the electrode surface there was a 143% increase to 0.172 cm^2^, with a linear relationship between the peak current (*I*_p_) of the reversible process and the square root of the scan rate, as shown in Fig. 4(a). Nanoparticles serve as ideal matrices for enzyme immobilization due to their capacity to promote enzyme adsorption, loading, and wiring.^49^ Using AuNPs to modify electrodes can significantly increase the electrode's ECSA, thereby enhancing the physical adsorption of GOx on the electrode and improving the sensor's stability. Furthermore, AuNPs have excellent electrical conductivity, facilitating electron transfer between GOx and the base layer of the electrode, which will further improve the sensitivity of the sensor. This step makes the electrode surface favourable for both GOx immobilisation and charge transport and increases the long-term stability of the sensor.^49–51^

To validate the performance of this sensor, the detection of glucose was performed in both PBS buffer and artificial urine. When the sensor was exposed to glucose, GOx on the sensor surface reacted with the glucose to produce H_2_O_2_, which was then reduced by Prussian blue to result in a change in current.^52^ As shown in Fig. 4(b) and (c), both detections in buffer solutions and artificial urine showed linear correlations between the reduction currents and the glucose concentration ranging from 50 to 1000 μM, with RSD values below 8.5% and 5.3%, respectively. The representative time *vs.* current results are shown in Fig. S11.† The sensitivity of the glucose sensor in PBS buffer and artificial urine, therefore, were found to be 21.3 μA cm^−2^ mM^−1^ (1.5 μA mM^−1^, *R*^2^ = 0.999) and 17.9 μA cm^−2^ mM^−1^ (1.3 μA mM^−1^, *R*^2^ = 0.998), with the corresponding LODs of 30 μM and 60 μM, respectively. Such differences in sensitivity could possibly be caused by interference from compounds in the artificial urine, suggesting that further steps are required to determine the selectivity of the glucose sensor.

The selectivity of our paper-based glucose sensor was studied by detecting glucose and a number of commonly found interferents in urine, namely glycine, urea, sarcosine, creatinine, uric acid, albumin, and ascorbic acid,^53,54^ as shown in Fig. 4(d). The maximum signal change, which came from uric acid, is only 5.8% of the glucose detection signal, indicating the excellent specificity of our glucose sensor. Then, the long-term stability of this sensor was tested by taking measurements every week for 4 weeks, with the storage conditions at 4 °C in sealed Petri dishes. The response of the glucose sensor after 4 weeks maintained 82.68% of the initial response with an RSD of 3.99%, which demonstrates good long-term stability and indicates the potential to be used for accurate and consistent measurements over time, as shown in Fig. 4(e). To characterise the reusability, the same sensor was used repeatedly to measure 1000 μM glucose in both PBS buffer and artificial urine for ten measurements. As shown in Fig. 4(f) and (g), the RSD of the current response was maintained at 2.52% and 2.27% in PBS and artificial urine, respectively, after ten repetitions. Lastly, three glucose sensors fabricated using identical methods from different batches were used to confirm their reproducibility, as shown in Fig. 4(h) and (i). The correlation between their linear fittings were highly overlapping, indicating an excellent reproducibility of the proposed fabrication methods.

### 3.5. POC analysis of urinary pH and glucose in clinical samples

To ensure that this dual functional sensing platform is practically useful, its performance was then validated in urine samples from both CKD patients and healthy individuals. This study received ethical approval from the national research ethics committee (23/NW/0230), which allowed for the collection of urine samples from anonymous patients with renal disease. Healthy individuals were collected in line with the UK NHS national research ethics guidelines from clinical service development, with all samples appropriately anonymised. We collected urine clinical samples from seven CKD patients (CS1 to CS7) and two healthy individuals (CS8 and CS9). For each sample tested, three repeats were performed in parallel.

**Fig. 5:**
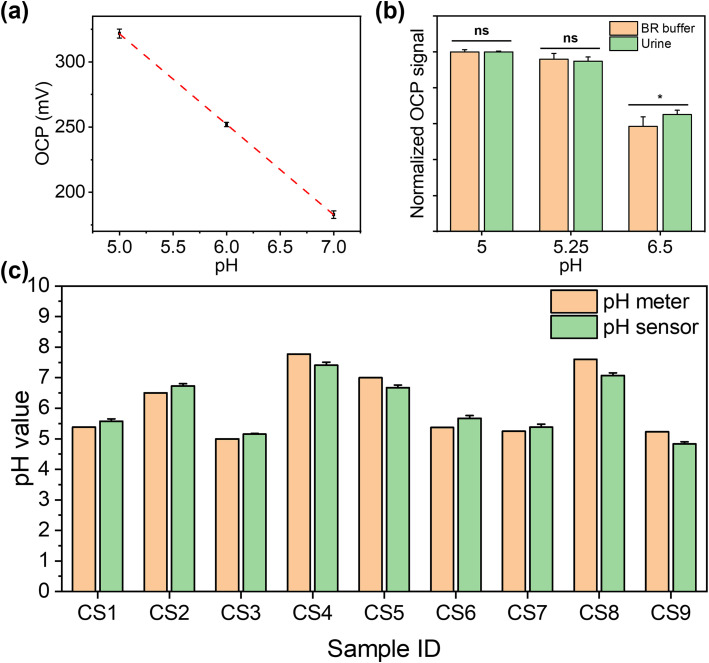
Detection of pH in human urine using a pH sensor. (a) Calibration curve of the pH sensor in healthy individual urine samples (*n* = 3). (b) Signal intensity comparison between the tests in BR buffer and urine samples for the same pH level (*n* = 3, ns = *p* > 0.05, *p < 0.05). (c) pH value of urine measured by the pH sensor in comparison to a commercial pH meter as a reference (CS1–CS7 are from CKD patients, CS8 and CS9 are from healthy individuals).

For pH determination, the pH of healthy individual urine sample was adjusted using 0.1 M NaOH and 0.1 M HCl to calibrate the pH sensor before the first measurement. The measured pH values in human urine samples were cross-validated against the results from commercial pH meters in UK quality assurance chemical pathology laboratories. Fig. 5(a) shows the calibration curve of the pH sensor in urine between the range of 5.0 and 7.0 (obtained *via* a commercial pH meter), which demonstrated a good linear response (*y* = −69*x* + 668, *R*^2^ = 0.99). We further compared the signal intensity in BR buffer and urine with the same pH value, as shown in Fig. 5(b). As shown in Fig. 5(c), we compared the pH results measured using commercial pH meters and our pH sensors. The results from our sensor show no significant difference with the results from the commercial pH meter in urine (*p* > 0.05, as determined by *t*-test; Pearson correlation coefficient: 0.96). This shows the excellent selectivity of our sensor for the detection in human urine, as the interference from non-target substances is insignificant. The detailed results are shown in Table S2,† with RSD values between 0.51% and 4.17%. While our data are consistent with those from a commercial pH meter, however, instances of “false negative” and “false positive” cases were observed. This indicates that reliance on pH values exclusively as a diagnostic indicator for CKD is insufficient. Consequently, it necessitates the concurrent assessment of additional biomarkers, *e.g.* glucose, to enhance diagnostic accuracy. This approach aligns with the prevailing consensus within the medical community regarding CKD diagnosis methodologies.

**Fig. 6:**
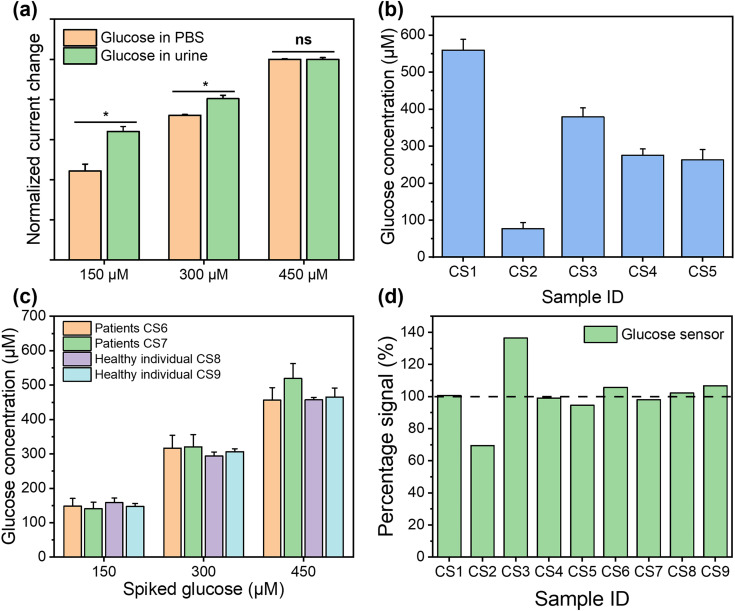
Detection of glucose in clinical samples. (a) Signal intensity comparison between the tests in PBS and spiked healthy individual (CS8) urine samples for the detection of the same glucose concentrations (*n* = 3). (b) Glucose concentrations measured from 5 CKD patient samples using electrochemical sensors (*n* = 3). (c) Glucose concentrations measured from CKD samples (CS6, CS7) and healthy individuals (CS8, CS9) using the spike recovery method at 150 μM, 300 μM, and 450 μM, respectively (*n* = 3; all *p*-values > 0.05, with CS6 as the reference). (d) Signal percentage of glucose concentration in urine measured by the glucose sensor in comparison to dipstick methods as a reference (CS1–CS7 are from CKD patients, CS8 and CS9 are from healthy individuals; samples CS6 to CS9 were spiked with 300 μM glucose).

For the healthy individuals, their glucose concentration in urine should be close to 0. The maximum detectable concentration of our glucose sensor is 1000 μM. When the glucose concentration is found to exceed the maximum detection range, the patients need to seek immediate medical intervention, where the absolute concentration in a frontline test is less important. To characterise the performance of the glucose sensor in clinical samples, where glucose concentration exceeds the maximum measurement range, the samples were diluted with PBS buffer for analysis. Dipstick has currently been used as the gold standard method for urinary glucose analysis in frontline clinical settings. Different concentrations of glucose were detected from samples CS1 to CS5, while it is absent from CS6 to CS9, using dipstick methods, as shown in Fig. S12.† For CKD patients, their renal function for glucose reabsorption gradually declines. When their plasma glucose concentration exceeds the renal reabsorption capacity, only the excess glucose will be excreted through the urine.^55,56^ It can be seen that glucose was not found in CS6 and CS7, which may be due to the fact that the patients were at the early stages of CKD and their kidneys were able to reabsorb glucose more efficiently despite the impaired kidney function, resulting in the glucose concentration below LOD. In addition, the patient's dietary habits and drug use could directly affect the glucose concentration in urine at the sampling time point. pH and glucose are two independent biomarkers for CKD, which can be affected by both daily food intake and other underlying conditions. The advantage of simultaneous analysis of both biomarkers in this work is to allow the crosscheck between the conclusions from two biomarkers to mitigate “false negatives” and “false positives”. After this preliminary POC test, the at-risk individual (indicated by either or both biomarkers) can be referred for more complicated tests. To support this strategy from a technical aspect, we could further improve the sensor design by including multiple pH and glucose sensors on each device to enhance the diagnostic accuracy statistically, while the cost increase is negligible. For the electrochemical analysis, the patient samples were diluted 50 times using PBS buffer to ensure that the glucose concentrations fall in the measurable range of our sensor. The healthy individual urine samples (CS8 and CS9) and two patient samples (CS6 and CS7), where glucose was not detected, were analysed using spike recovery method. We firstly compared the detection of the same glucose concentrations in PBS solution and spiked healthy individual urine samples, as shown in Fig. 6(a). The signal intensities from two sets of measurements shows comparable trends, which indicates the excellent selectivity of our sensor, as it suffers from little matrix effect in the urine sample. For the five samples (CS1–CS5), although the electrochemical sensor shows more accurate results, which make them quantitatively incomparable to the interval readings from dipsticks, their results follow the same trend and roughly fell in the same ranges, as shown in Fig. 6(b). For the samples in which glucose were not detected (CS6–CS9), these were then retested by spiking the sample with a glucose concentration of 150 μM, 300 μM, and 450 μM, as shown in Fig. 6(c). The signal intensities from four samples were highly consistent with the same glucose concentration with *p* values > 0.05, indicating the excellent accuracy and stability of the sensors. In Fig. 6(d), we compared the results from the electrochemical sensor to those from the dipstick method, where the latter were normalised to 100%. The degree of mismatch between the two sets of results largely depends on the difference between the real concentrations of glucose in clinical samples and the nearest colorimetric reference. The detailed results of glucose detection from human urine samples are summarised in Table S3.† We compared the detection of glucose in clinical samples using this sensor platform and the standard method, with a Pearson correlation coefficient calculated as 0.98, indicating that this platform has competitive performance to the commercial method. The recoveries of glucose from human urine samples varied from 69.45% to 136.45% (RSD between 0.82% and 5.37%), which is because the dipstick method has a limited number of concentrations on the colorimetric reference card for comparison. This indicates that dipstick is unsuitable for quantitative analysis. In addition, other factors such as ambient light and urinary colorants could potentially have introduced errors. These results show that our glucose sensor is able to provide similar qualitative POC analysis compared to the gold standard method in frontline clinical settings, with potential for more quantitative analysis for accurate concentration-dependent disease progression tracking. The successful clinical validation demonstrated the potential of our dual functional biosensor as a rapid, accurate, safe, and user-friendly POC platform for the analysis of urine biomarkers in frontline clinical settings.

Table 2 summarises the state-of-the-art technologies for pH and glucose detection. Compared with these technologies, our pH sensor exhibited a sensitivity of 71.58 mV pH^−1^ and LOD of 0.07 pH in the 4.0–9.0 pH range, whilst the glucose sensor showed sensitivity of 21.3 μA cm^−2^ mM^−1^ and LOD of 30 μM within the 50–1000 μM range, satisfying the requirements for clinical urine analysis. In addition to the competitive performance, our biosensor platform has been well integrated with the urine sampling vials, which significantly reduce the health risk of sample splash to practitioners and simplify the analysis operations.^8^ Overall, our biosensor platform, validated in human urine samples, shows obvious advantages in on-site testing, operational complexity, and reduced risk of sample splash.

**Table 2:** Comparison of this work with previously reported biosensors

Detection	Active material	Sensitivity	Range	LOD	Real samples	Risk level (sample splash)^a^	Potential in POC^b^	Ref./brand
pH sensor	Thymol blue/methyl red/phenolphthalein	—	6.0–8.0	0.5	Sweat	++	++	57
pH sensor	Graphitized mesoporous carbon/polyaniline	58 mV pH^−1^	2.0–11.0	—	Urine/saliva	++	+++	58
General pH test strips	Indicator dyes	—	0–14.0	1.0 pH	Body fluids	+++	+++	Simplex Health
Narrow range pH test strips	Indicator dyes	—	5.5–8.0 pH	0.2 pH	Urine/saliva	+++	+++	Hydrion
pH meter	Hydrogen ion selective electrode (HISE)	—	0–14.0	0.01 pH	Body fluids	++	+++	FiveEasy
Blood gas analyser for pH measurement	HISE		6.3–8.0	0.001 pH	Blood	+	+	Radiometer
pH sensor	IrOx	71.58 mV pH^−1^	4.0–9.0	0.07 pH	Urine	+	+++	This work
Glucose sensor	GOx/CNTs/Ti_3_C_2_T_*x*_/PB	35.3 μA cm^−2^ mM^−1^	10–1500 μM	0.33 μM	Sweat	++	+++	59
Glucose sensor	AuNPs/graphene	0.044 mA/log(M)	10–10 000 μM	6.3 μM	—	++	+++	60
Blood glucose meter	GOx/glucose dehydrogenase (GDH)	—	1100–33 300 μM	100 μM	Blood	+++	+++	Sinocare
Urine glucose test strips	GOx/peroxidase	—	0–110 000 μM	5600 μM	Urine	+++	+++	One Step
Glucose sensor	GOx/PB/AuNPs	21.3 μA cm^−2^ mM^−1^	50–1000 μM	30 μM	Urine	+	+++	This work

a Risk level is determined by the following: (1) whether the sample is exposed to air during testing, (2) the duration of exposure of the sample to air, and (3) the risk of sample splashing during the process.

b Potential in POC is determined by the following: (1) whether the sensor is flexible or not, (2) the volume of the sensor, (3) the ease of use and readability of the sensor, (4) the accuracy and sensitivity of the sensor, and (5) the storage and shelf life of the sensor.

## 4. Conclusions

Urinary biomarkers, such as abnormal pH values and glucose concentrations, are associated with the development of CKD. We demonstrate a paper-based dual functional electrochemical sensor for the analysis of urinary pH and glucose, which can be integrated into the urine sampling vial widely used by the NHS. While the sensors show competitive performance with other state-of-the-art research, our analysis platform distinguishes itself through a pioneering fusion with urine sampling vials. This inventive integration not only provides a platform with competitive sensing performance for urine analysis but also introduces a significant advancement in reducing exposure risks for frontline clinical practitioners, marking a dual achievement in both technological innovation and occupational safety. The paper-based platform is compatible with the current large-scale device production techniques, which ensures its cost-effective production for POC analysis. Electrochemically deposited IrOx under optimal conditions has been used as the pH-sensitive layer, while the GOx and Prussian blue pair has been used for glucose sensing, with the synergic effect from AuNPs and the graphite/chitosan framework. In the clinical validations, this dual functional sensing platform has shown competitive performance to that of the commercial gold standard technologies while significantly reducing the health risk to the frontline practitioners and simplifying the diagnostic procedures. For the prospective commercial application of this sensing platform, there are several possible solutions to enable long-term use. For example, the calibration samples can be provided to users before performing each test, or the performance decay curve could be provided from the manufacturer and integrated into the analyses software for benchmarking, thus allowing users to obtain accurate readings after long-term storage. Our findings could inspire future designs of POC biosensors for scalable multiplexed screening of biomarkers in complex media beyond urinalysis, such as for wastewater-based epidemiology^61,62^ and early warning and surveillance for zoonotic diseases.^63^

## Author contributions

Y.-J. L. was involved with conceptualization, data curation, formal analysis, investigation, validation, and visualization and wrote the original draft. Y. K. was involved with the methodology. Y. H. was involved with writing, reviewing, and editing the final manuscript. Y.-X. L. was involved with the methodology. R. A. was involved with the methodology. W. Z. was involved with providing resources. B. D. B. was involved with writing, reviewing, and editing the final manuscript. A. K. Y. was involved with writing, reviewing, and editing the final manuscript. A. D. was involved with providing resources and writing, reviewing, and editing the final manuscript. T.-C. L. was involved with writing, reviewing, and editing the final manuscript. B. L. was involved with conceptualization, supervision, providing resources, and funding acquisition, wrote the original draft, and wrote, reviewed, and edited the final manuscript.

## Conflicts of interest

There are no conflicts to declare.

## Supplementary Material

LC-024-D4LC00163J-s001

LC-024-D4LC00163J-s002

LC-024-D4LC00163J-s003

LC-024-D4LC00163J-s004

LC-024-D4LC00163J-s005

LC-024-D4LC00163J-s006

LC-024-D4LC00163J-s007

LC-024-D4LC00163J-s008

LC-024-D4LC00163J-s009

LC-024-D4LC00163J-s010

LC-024-D4LC00163J-s011

LC-024-D4LC00163J-s012

LC-024-D4LC00163J-s013

LC-024-D4LC00163J-s014
