# Supplementary figures and images for: A paper-based dual functional biosensor for safe and user-friendly point-of-care urine analysis
Source: Lab Chip. 2024 Apr 12;24(9):2454–67. doi: 10.1039/d4lc00163j (PMC11060138; doi:10.1039/d4lc00163j)

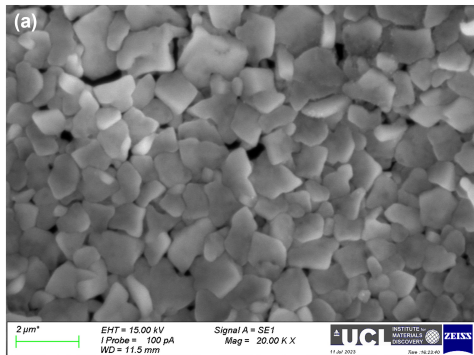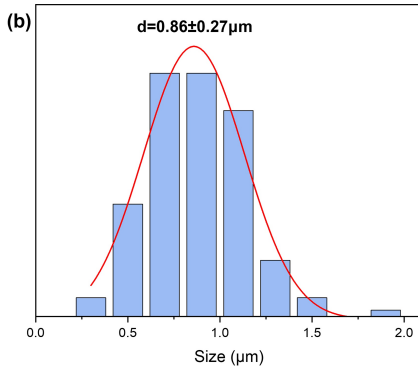

Supplement: LC-024-D4LC00163J-s002 [file LC-024-D4LC00163J-s002.pdf]

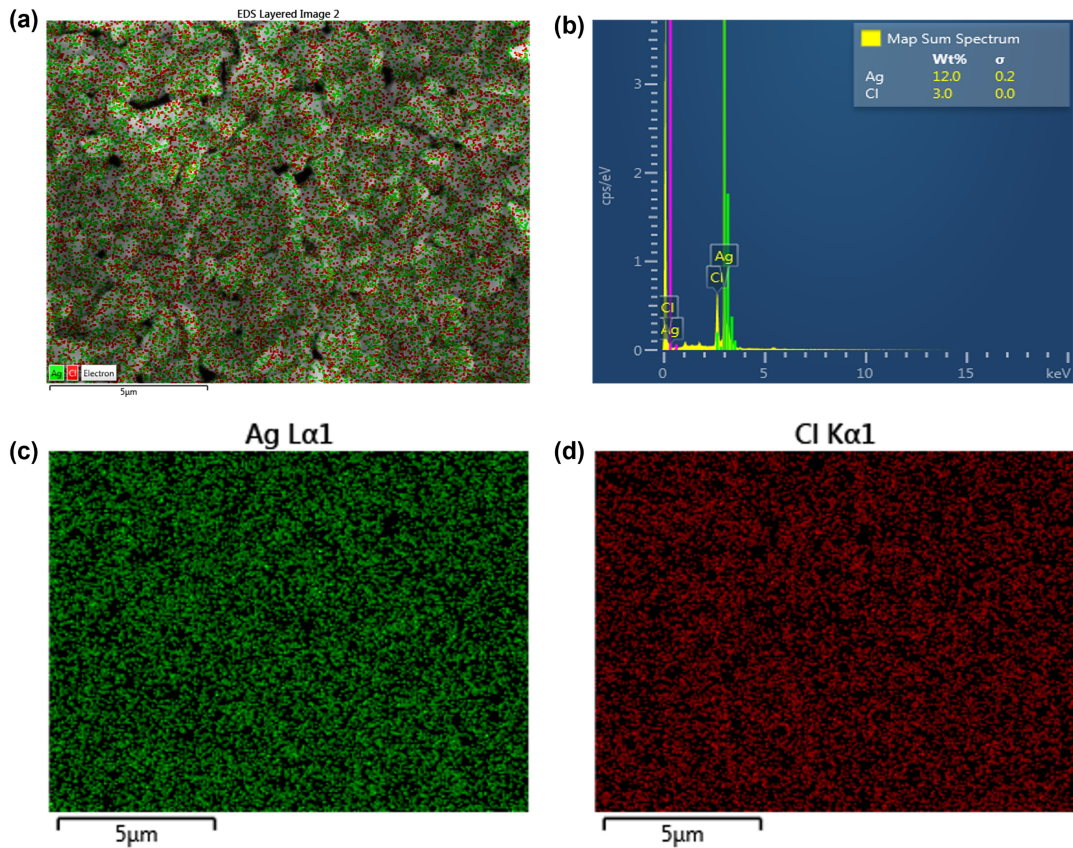

Supplement: LC-024-D4LC00163J-s003 [file LC-024-D4LC00163J-s003.pdf]

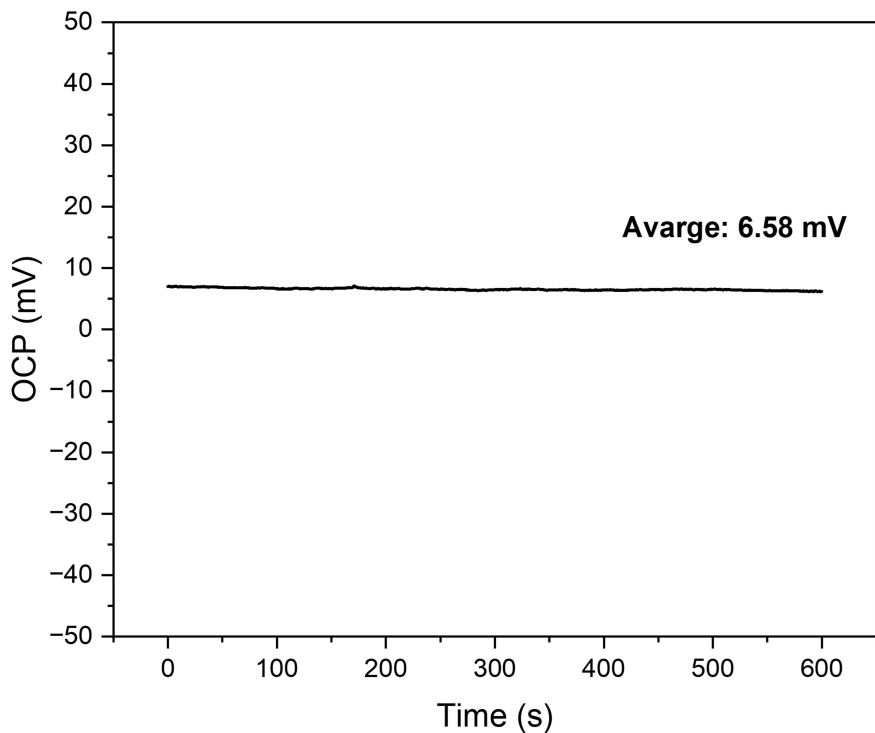

Supplement: LC-024-D4LC00163J-s004 [file LC-024-D4LC00163J-s004.pdf]

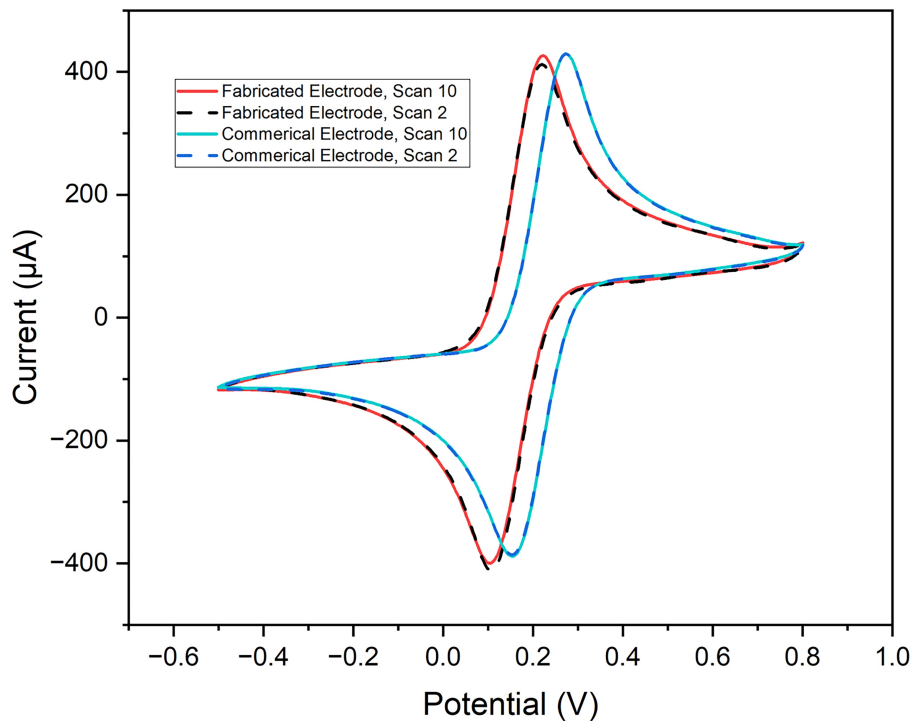

Supplement: LC-024-D4LC00163J-s005 [file LC-024-D4LC00163J-s005.pdf]

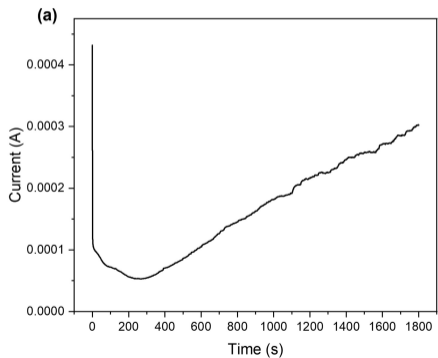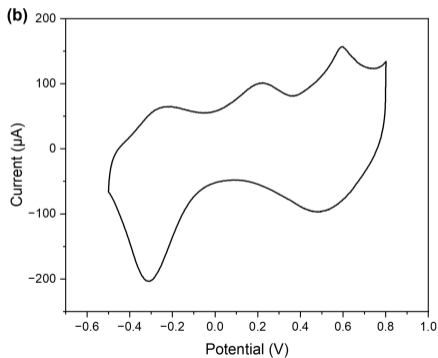

Supplement: LC-024-D4LC00163J-s007 [file LC-024-D4LC00163J-s007.pdf]

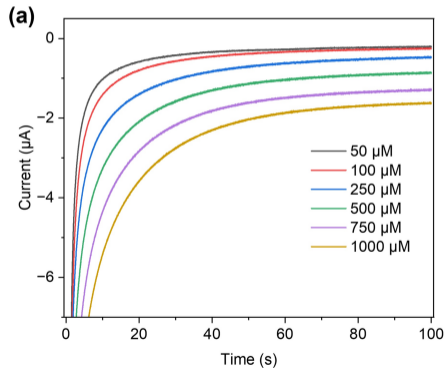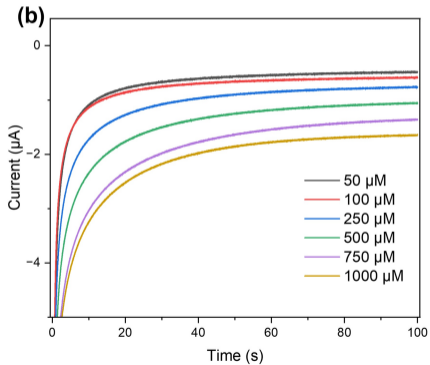

Supplement: LC-024-D4LC00163J-s009 [file LC-024-D4LC00163J-s009.pdf]

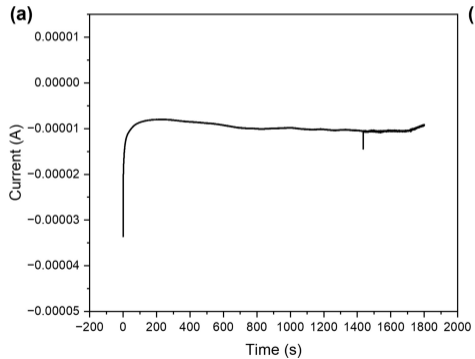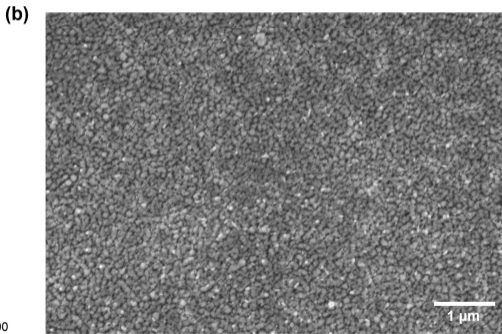

Supplement: LC-024-D4LC00163J-s011 [file LC-024-D4LC00163J-s011.pdf]

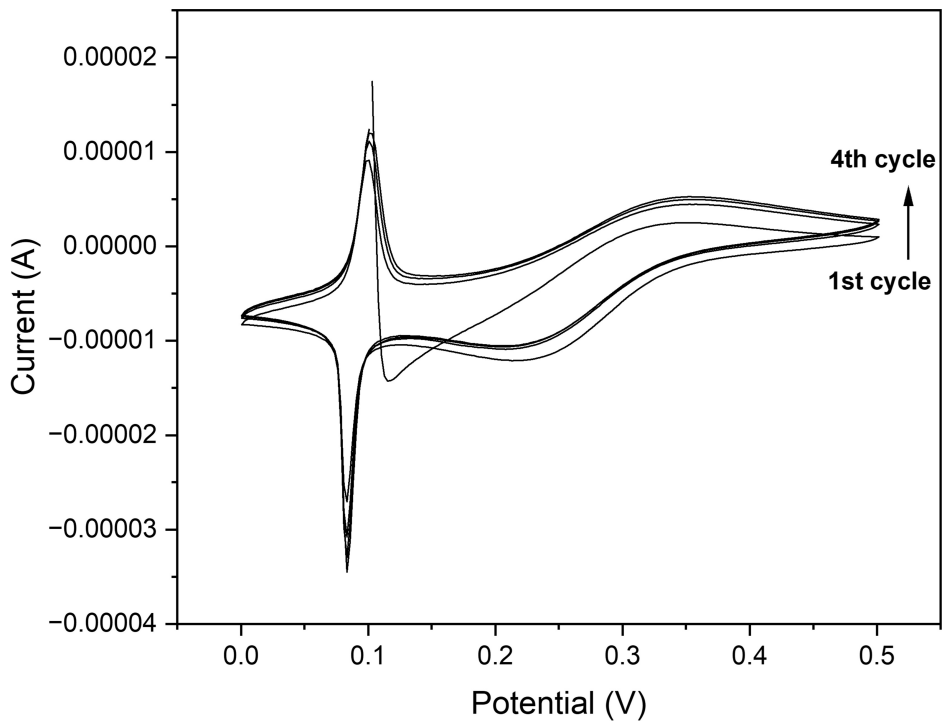

Supplement: LC-024-D4LC00163J-s012 [file LC-024-D4LC00163J-s012.pdf]

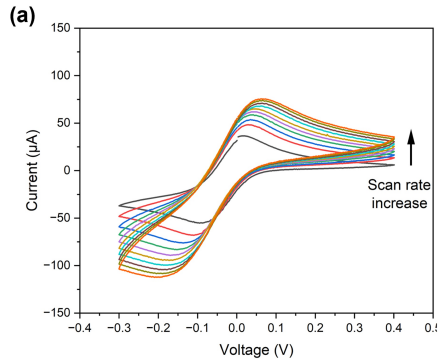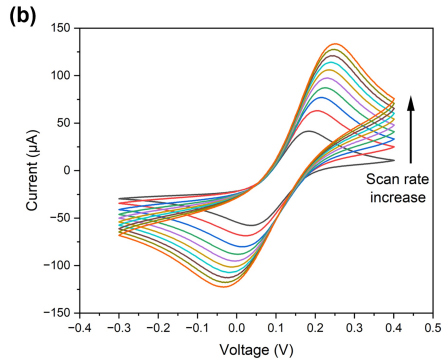

Supplement: LC-024-D4LC00163J-s013 [file LC-024-D4LC00163J-s013.pdf]

(a)

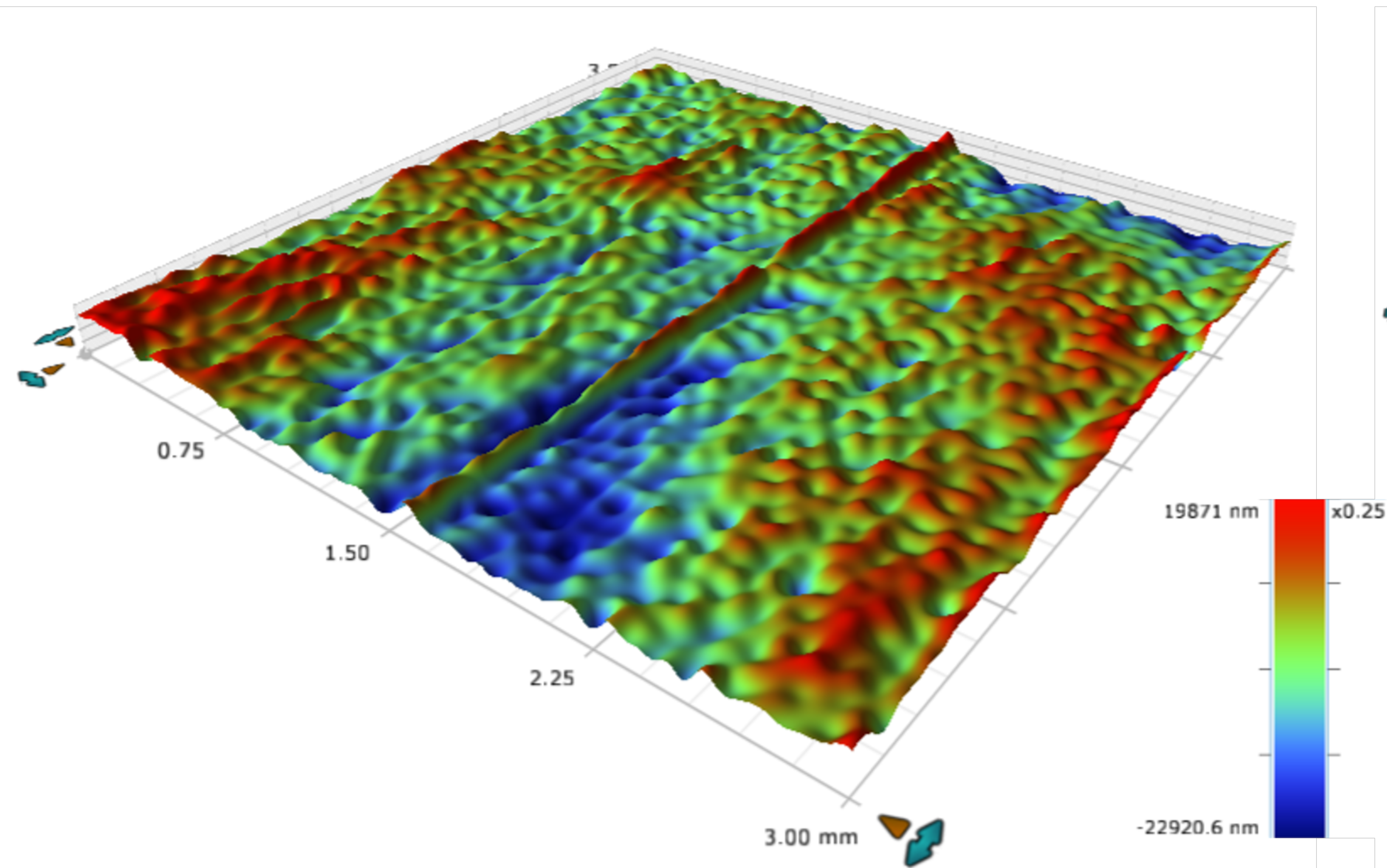

(b)

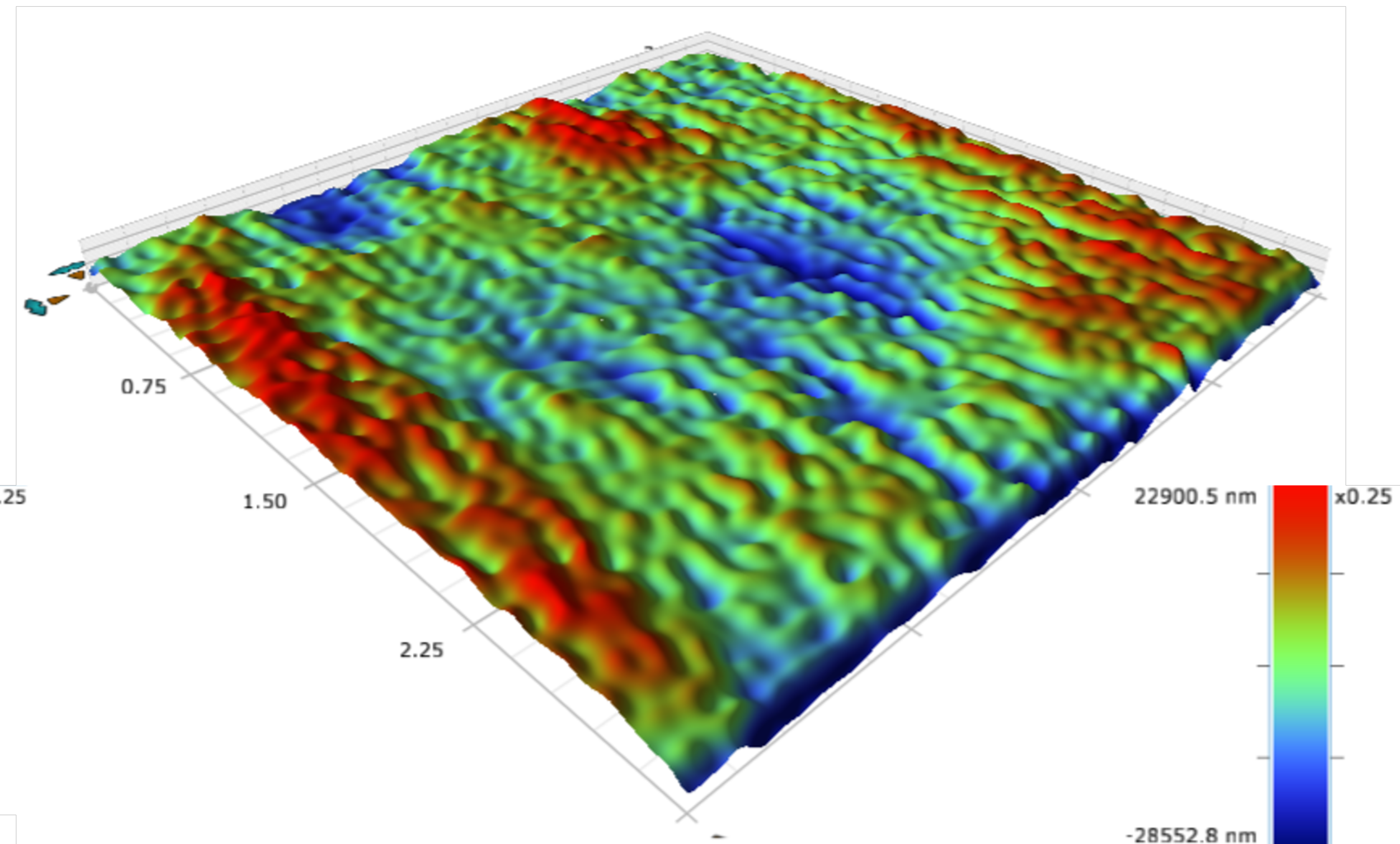

Supplement: LC-024-D4LC00163J-s014 [file LC-024-D4LC00163J-s014.pdf]
