# Supplementary material for: A paper-based dual functional biosensor for safe and user-friendly point-of-care urine analysis
Source: Lab Chip. 2024 Apr 12;24(9):2454–67. doi: 10.1039/d4lc00163j (PMC11060138; doi:10.1039/d4lc00163j)
Supplement: LC-024-D4LC00163J-s006 [file LC-024-D4LC00163J-s006.pdf]

Electronic Supplementary Material (ESI) for Lab on a Chip.
This journal is © The Royal Society of Chemistry 2024

Supplementary Information

A Paper-based Dual Functional Biosensor for Safe and User-Friendly Point-of-Care Urine Analysis

Yujia Li [a, b], Yingqi Kong [a, b], Yubing Hu [c], Yixuan Li [a, b], Rica Asrosa [a, b, d], Wenyu Zhang [e], Buddha Deka Boruah [a], Ali K. Yetisen [c], Andrew Davenport [f], Tung-Chun Lee [a], Bing Li [a, *]

[a] Institute for Materials Discovery, University College London, London, WC1E 7JE, UK

[b] Department of Chemistry, University College London, London, WC1E 7JE, UK

[c] Department of Chemical Engineering, Imperial College London, London, SW7 2AZ, UK

[d] Department of Physics, Faculty of Mathematics and Natural Science, Universitas Sumatera Utara, Medan 20155, Sumatera Utara, Indonesia

[e] Department of Materials, University of Oxford, Parks Road, Oxford, OX1 3PH, UK

[f] UCL Department of Renal Medicine, Royal Free Hospital, University College London, Rowland Hill Street, London, NW3 2PF, UK

Cost Analysis

This sensor platform requires the design and production of a reusable portable digital readout system, which is expected to be ~£20, similar to the commercial portable blood glucose meters. The materials costs for manufacturing these pH and glucose sensors are expected to be ~£18.24 for 100 units, with a breakdown of the key fabrication processes in Table S1. It is worth noting that the sensor fabrication processes are compatible with the current industrial methods, which would further lower the cost for the large-scale commercial production.

Table S1. Material cost analysis of manufacturing 100 sensor platforms

**Manufacturing process**	**Cost (£)**
Fabrication of paper-based electrodes	2.27
Fabrication of pH sensor	3.10
Fabrication of glucose sensor	7.61
Clinical urine sampling vial	5.26
**In total**	18.24

Preparation and characterisation of IrOx modified electrodes

Chronoamperometric method has been used to fabricate the IrOx layer on the Au electrode. Firstly, $IrCl_4$ (0.15 g) was dissolved in DI water (100 mL) with the magnetic stirring for 30 mins. Then, H_2O_2 (1 mL, 30 wt%) and $(COOH)_2 \cdot 2H_2O$ (0.5 g) were consecutively added into above solution and followed by a 10 mins stirring. K_2CO_3 was used to adjust the solution pH to 10.5, which allow the reaction to occur. The resulting solution was kept in dark for 48 h to stabilize, which is indicated by the colour change from yellow to light purple. Then the solution was stored in dark at 4°C when not in use. The home-made Au electrodes were immersed into this solution and 0.7 V was applied onto the working electrode for 30 mins, which results in a dark blue IrOx film on the electrode surface. Figure S1a shows chronoamperometric curve recorded in prepared deposit solution. In order to obtain a voltametric fingerprint of a deposit, the electrodes after the electroplating were transferred to a pH 7.40 PBS buffer solution and cyclic voltammograms were recorded. The voltammograms displayed the characteristic reversible redox behaviour of activated iridium electrodes [1], as shown in Figure S1b. This strongly suggests that a deposit on the electrode is the iridium oxide.

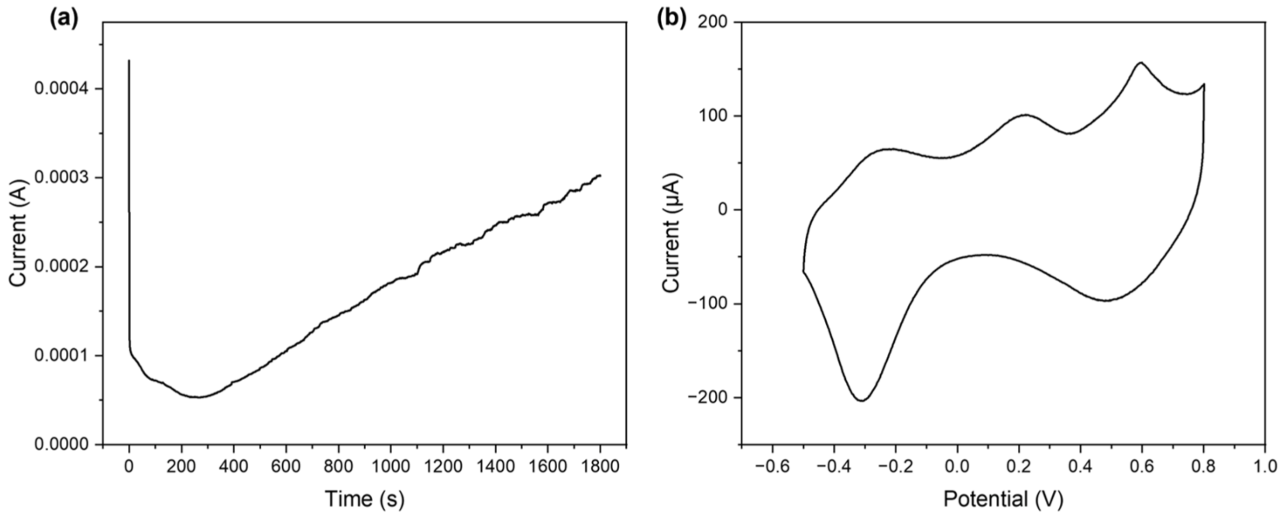

Figure S1. (a) Chronoamperometric curve (E = 0.7 V) of IrOx modified electrode. (b) CV of the IrOx modified electrode. Electrolyte: 0.2 M PBS buffer at pH 7.4 containing 0.15 M NaCl. Scan rate: 50 $mV \cdot s^{-1}$.

Preparation and characterisation of AuNPs modified electrodes

Chronoamperometric method has also been used to fabricated AuNPs modified working electrode (Figure S2a). The electrode was immersed into a 2 mM $HAuCl_4$ solution with 2 M H_2SO_4 as the solvent. Then a constant potential of -0.1 V was on the working electrode for 30 min for AuNP deposition, where an Au counter electrode and an Ag/AgCl reference electrode were used in three electrode system. From Figure S2b, it can be seen that the AuNPs prepared by electrodeposition are uniformly distributed on the electrode surface. AuNPs were used to shorten the distance between the electrode surface and the flavin adenine dinucleotide (FAD) of GOx and effectively increase ECSA of the electrode to accelerate electron transfer.

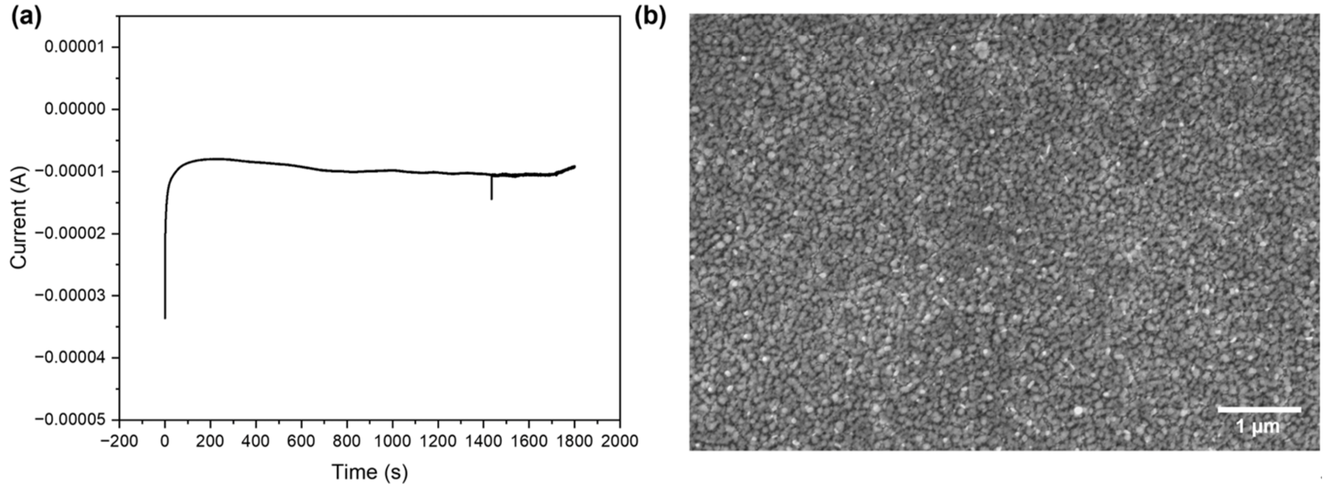

Figure S2. (a) Chronoamperometric response of AuNPs modified electrode. (b) SEM image of AuNPs on paper-based gold electrodes.

Preparation of Prussian blue modified electrodes

The Prussian blue layer was used as the H_2O_2 sensitive layer in this project. This layer was electrochemical deposited by immersing the AuNPs modified Au electrode into a 50 mL Prussian blue solution (100 mM KCl, 2.5 mM $FeCl_3$ and 2.5 mM $K_3Fe(CN)_6$ in 100 mM HCl) and running CVs from 0 to 0.5 V at a scan rate of 20 mVs^{-1} for 4 cycles. Au electrode and Ag/AgCl electrode were used as the counter electrode and reference electrode respectively. The results are illustrated in Figure S3. The pair of redox peaks is corresponding to the reduction and oxidation process between Prussian white and Prussian blue, which involves the exchange of potassium ion (Equation S1) [2]. The current peak located at 0.08 V is attributed to the reduction of Prussian white, while the current peak at 0.11 V is due to the oxidation of Prussian white to Prussian blue [3].

$$[K_2Fe(CN)_6Fe]_x - xK^+ - xe^- \leftrightarrow [KFe(CN)_6Fe]_x \qquad (S1)$$

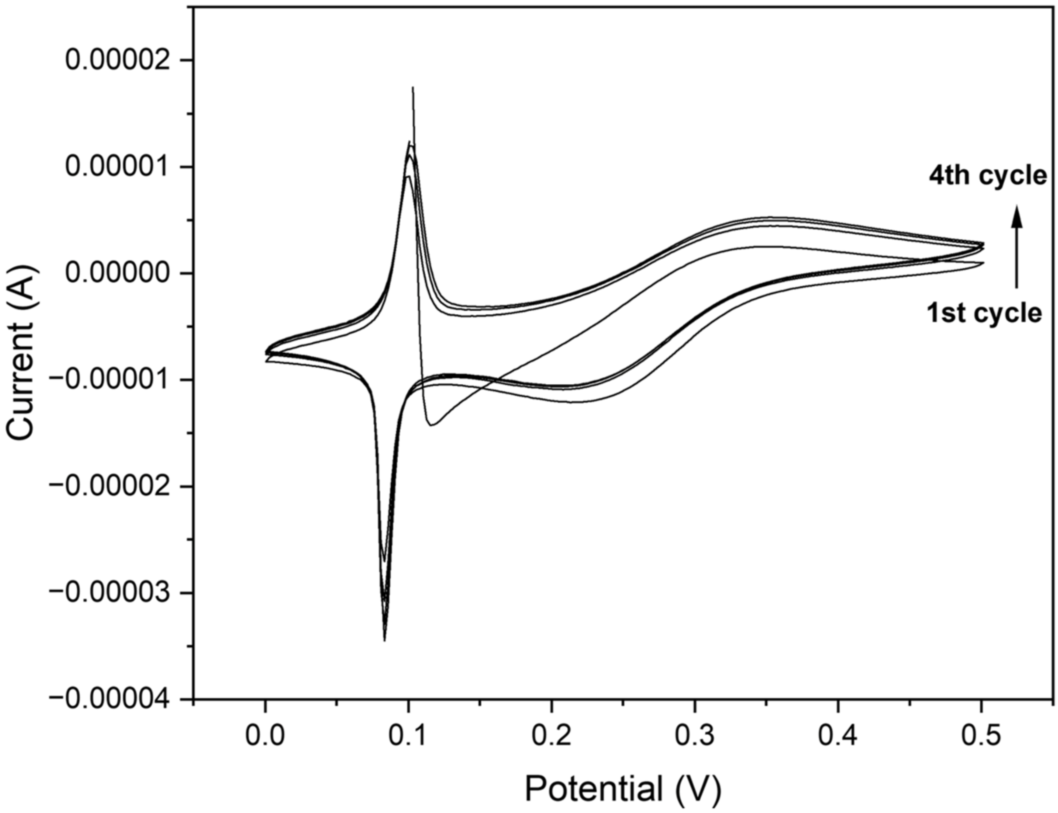

Figure S3. Cyclic voltammograms of Prussian blue modified electrode. Scan rate: 20 $mV \cdot s^{-1}$.

ECSA characterisation of AuNPs modified electrode

Electrochemical active surface area (ECSA) is an important parameter in electrode applications. In this project, the CV characteristics were used to determine the ECSA of bare Au electrodes and AuNPs modified electrodes in the electrolyte solution containing 10 mM $K_3Fe(CN)_6$, 10 mM $K_4Fe(CN)_6$, and 1 M KCl. The CV were running from 0 to 0.5 V at scan rates from 20 to 200 mVs^{-1} with bare Au or AuNPs modified electrode as the working electrode, Au electrode as the counter electrode, and Ag/AgCl electrode as the reference electrode. Both anodic and cathodic peak heights were proportional to the square root of the scan rate. These peak heights were used for further calculations of ECSA, which were estimated using Randle-Sevcik equation as 0.071 cm^2 and 0.172 cm^2 for bare Au and AuNPs modified electrodes, respectively.

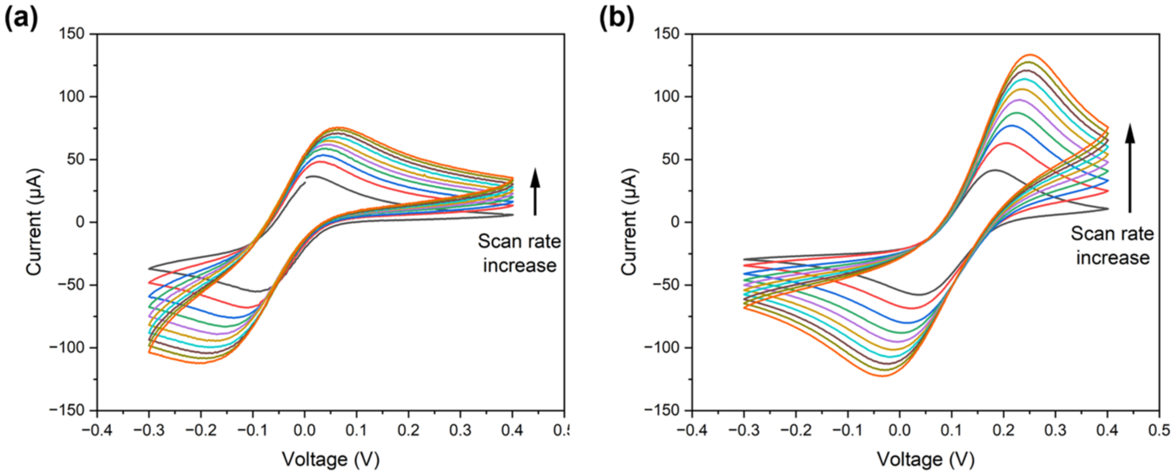

Figure S4. CV plot of (a) bare Au (b) AuNPs modified electrode with scan rate from 20 to 200 mVs^{-1}.

Surface characterisations of paper substrate and gold electrode

The surface morphology of paper substrate and Au electrodes would greatly affect the sensor's performance. The 15 nm Cr was first sputtered onto the paper substrate, then followed by 150 nm Au for working and counter electrodes, or 150 nm silver for reference electrode. Figure S5a showed the fibrous structure of the paper substrate, with an average surface roughness of 8.0 µm, which facilitates the bonding of the Cr and Au layers. As shown in Figure S5b, the fibrous structure of the paper substrate is maintained on the surface of the Au electrodes, which will in turn enhancing the adhesion of the electrodeposited AuNPs and IrOx.

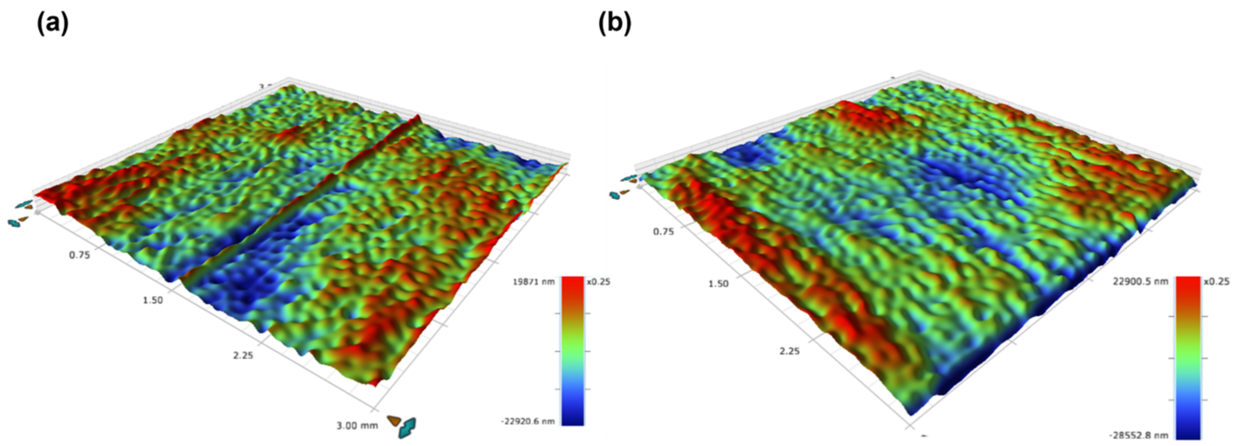

Figure S5. 3D mappings of (a) the paper substrate and (b) the Au electrode on paper substrate.

Preparation and characterisation of Ag/AgCl electrodes

In this study, we developed a miniaturised Ag/AgCl pseudo reference electrode. The silver layer with a thickness of 150 nm was sputtered onto the substrate. Then a 14 % NaClO aqueous solution was dropped onto the silver reference electrode to react for 1 min until the silver turns to a dark and then rinsed with DI water and blown dry with a nitrogen gun. The morphology of AgCl particles has a great effect on reference electrode's performance. From Figure S6a, which showed that AgCl particles were uniformly and firmly distributed on the electrode surface. The more uniform the particle distribution, the lower the current noise during use [4]. The particle size distribution was shown in Figure S6b, the average particle size of AgCl on the electrode is 0.86 μm, which helps to reduce the porosity of AgCl layer.

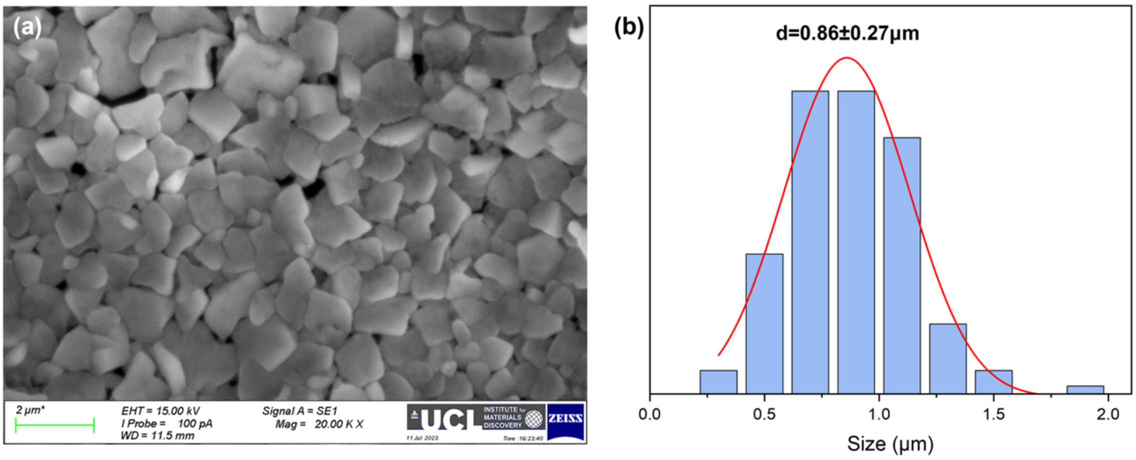

Figure S6. Characterisation of fabricated Ag/AgCl reference electrode. (a) SEM image of the Ag electrode after the AgCl layer formation. (b) Particle size distribution of AgCl.

EDS measurement of Ag/AgCl electrodes

Energy-dispersive X-ray spectroscopy (EDS) was used to examine the elemental composition and elemental distribution of the electrode. Figure S7a shown an elemental map matching Figure S6a, where Ag was shown in green and Cl in red. Figures S6c-d highlight the distribution of Ag and Cl elements on the fabricated Ag/Cl electrode. It can be seen that the electrode surface was uniformly covered by AgCl particles and a dense AgCl layer was formed, indicating that the electrode surface was effectively chlorinated. Figure S7b shows the EDS spectrum of AgCl, from which it can be known that the weight ratio of Ag to Cl elements is 4:1, it can be calculated that the ratio of Ag to Cl elements is 1.3:1. Theoretically, this value should be 1:1, the reason here might be that the Ag under the AgCl has been detected.

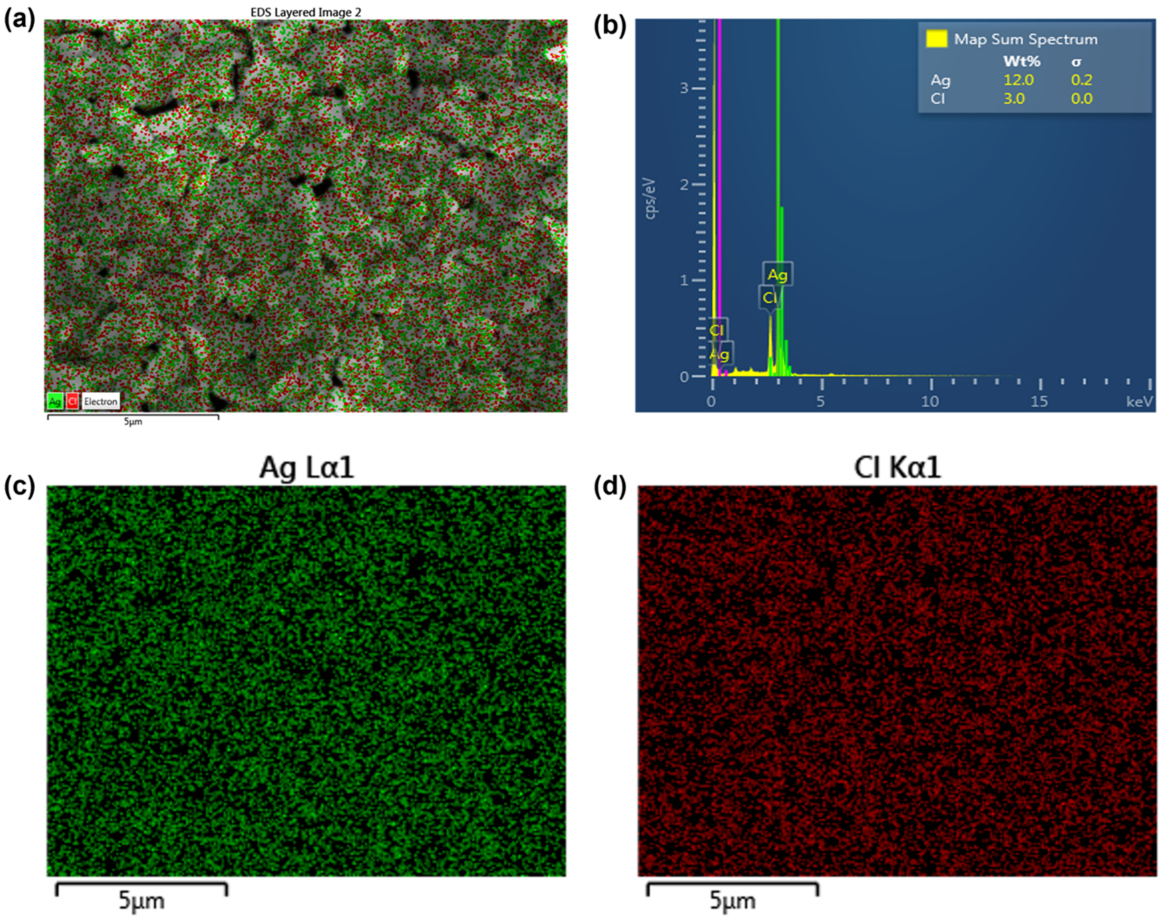

Figure S7. EDS of fabricated Ag/AgCl reference electrode. (a) EDS elemental map for Ag/AgCl. (b) EDS spectrum of Ag/AgCl. EDS elemental map for (c) Ag and (d) Cl.

Stability characterisation of Ag/AgCl electrodes

We compared the OCP of our fabricated Ag/AgCl reference electrode with that of a commercial Ag/AgCl electrode using a two-electrode system in 3.5 M KCl solution. Our fabricated Ag/AgCl electrode was used as the working electrode, and the commercial Ag/AgCl electrode as the reference electrode. OCP measurements were performed on electrochemical workstation (Autolab PGSTAT204, Metrohm, UK). The results shows that the potential difference between the fabricated and commercial electrodes, is 7.03 mV at 0 s and the average remains 6.58 mV in 10 minutes. Indicating that the Ag/AgCl reference electrodes fabricated in this project have excellent stability.

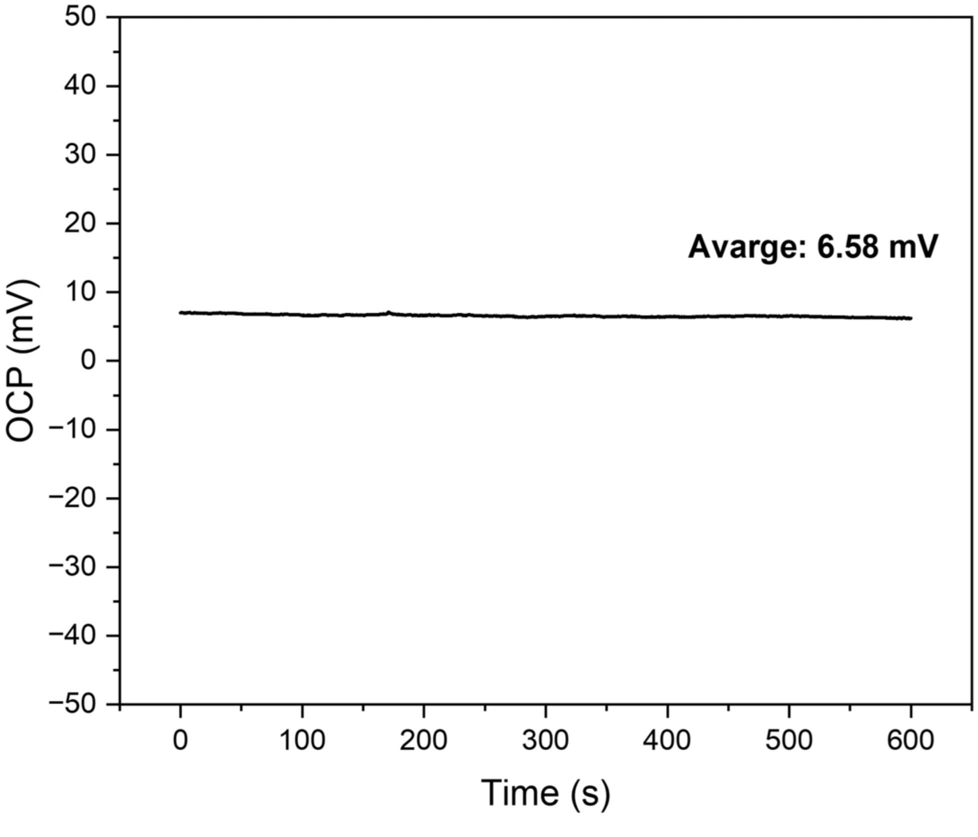

Figure S8. OCP of fabricated Ag/AgCl reference electrode in 3.5 M KCl solution

CV characterisation of Ag/AgCl electrodes

In order to further explore the difference between our fabricated Ag/AgCl reference electrode and commercial Ag/AgCl reference electrode. We compared the CV of our fabricated Ag/AgCl reference electrode with that of a commercial Ag/AgCl reference electrode using a three-electrode system in redox system $[Fe(CN)_6]^{3-/4-}$. As shown in Figure S9, both commercial and fabricated electrodes produce voltammograms with the same shape and intensity but different peak potentials. The peak potentials of home-made electrode did not change significantly from scan 2 to 10, indicating the excellent reversibility. The anodic (oxidation) and cathodic (reduction) peaks were observed to be shifted to the left by 52 mV and 49 mV respectively, in agreement with the results of Rohaized *et al.* [5], which corresponds to the potential difference between home-made Ag/AgCl electrode and commercial Ag/AgCl electrode. This is another evidence of the home-made Ag/AgCl electrode stable operation in solutions containing $[Fe(CN)_6]^{3-/4-}$. Indicating that our fabricated Ag/AgCl reference electrode behaves similarly with the commercial reference electrode.

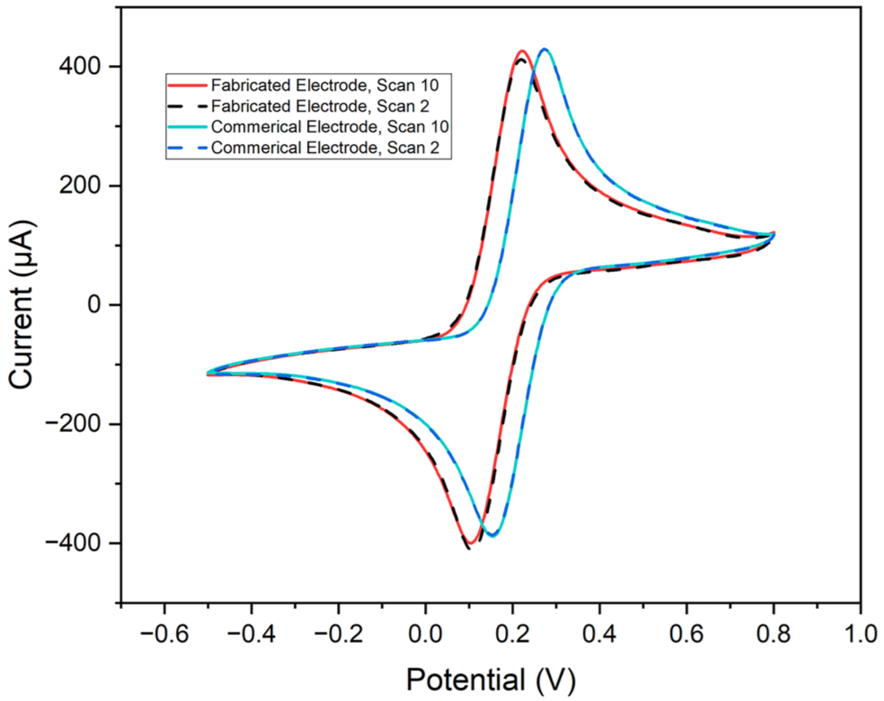

Figure S9. CV characteristics of home-made and commercial Ag/AgCl reference electrode in 10 mM $[Fe(CN)_6]^{3-/4-}$.

Electrochemical deposition of Prussian blue, drop-coat enzyme layer and Nafion layer

Prussian blue was electrochemically deposited onto the AuNPs-modified electrode via CV (from 0 V to 0.5 V, scan rate 20 mVs^{-1}, 4 cycles), followed by the drop-casting of 0.5 μL of GOx/BSA/chitosan/graphite composite, 0.5 μL glutaraldehyde (2 wt%), and 0.8 μL Nafion (0.5 wt%), respectively. As shown in Figure S10a, Prussian blue is uniformly distributed on the electrode surface. After drop-casted the enzyme solution, it shows a film which covered the electrode surface (Figure S10b). From Figure S10c, Nafion drop-casted electrode presents the similar appearance, which could protect the GOx from peeling off during the test.

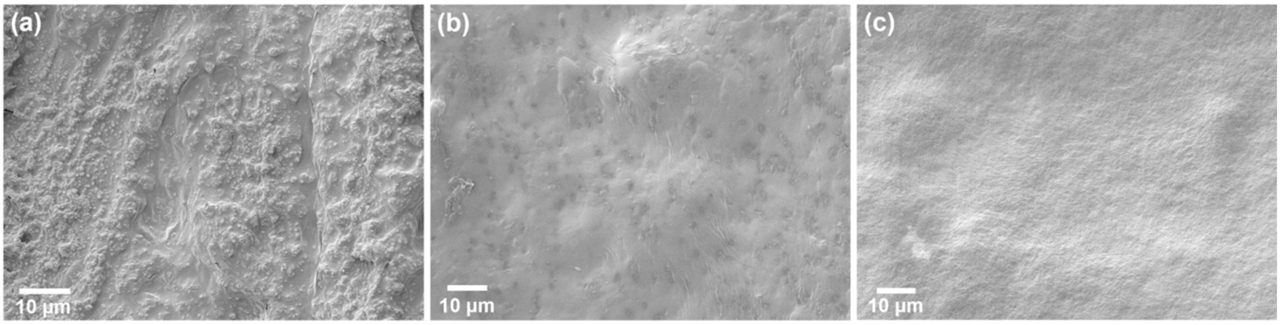

Figure S10. SEM images of (a) Prussian blue coated AuNPs-modified gold electrode; (b) GOx/BSA/chitosan/graphite composite on electrode; and (c) Nafion coated electrode.

Timed current response of the glucose sensor

We have characterised the glucose sensor in PBS buffered solution and artificial urine. The response of the glucose sensor was recorded using electrochemical workstation at a constant potential of -0.1 V for 100 seconds relative to an Ag/AgCl reference electrode. When the sensor is exposed to glucose in solution, GOx reacts with the glucose to produce hydrogen peroxide (H_2O_2), which is reduced by Prussian blue, resulting in a change in current [6]. The corresponding time currents of the glucose sensor for increasing glucose concentrations from 50 to 1000 µM are shown in Figure S11.

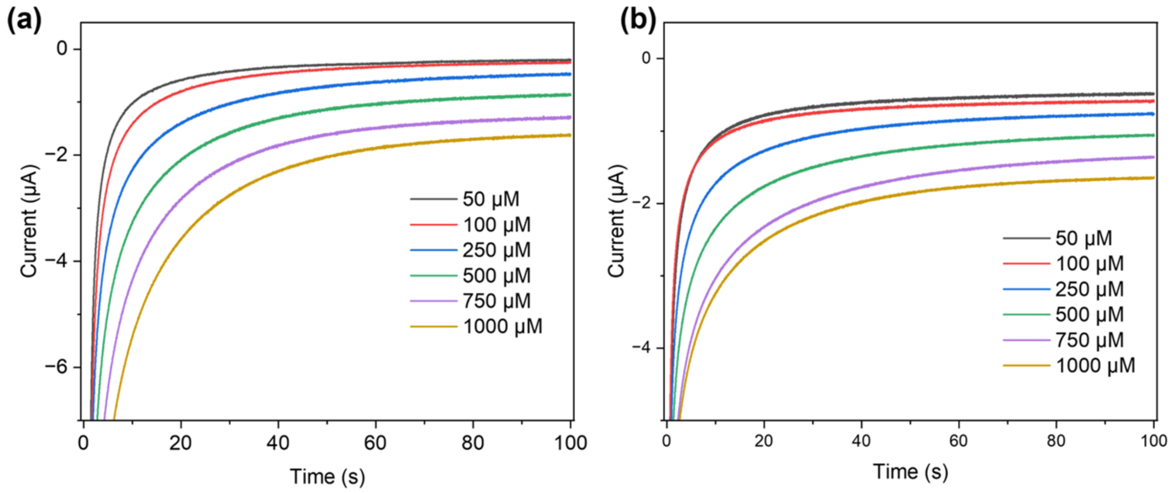

Figure S11. Timed current response of the glucose sensor in (a) PBS solution (b) artificial urine with increasing concentration of glucose (50-1000 µM). Operate voltage: -0.1 V, pH 7.5, 25 °C.

Glucose tests in human urine via commercial glucose detection kit

To validate the performance of this dual function biosensor for urine analysis against the dipstick method, the tests were performed in human urine samples from both healthy individuals and CKD patients sourced from Royal Free Hospital. As shown in Figure S12, different concentrations of glucose were detected in the urine of CKD patients and no glucose was detected in the urine of healthy individuals via dipstick method. This also illustrates the importance of monitoring glucose levels in urine for CKD patients.

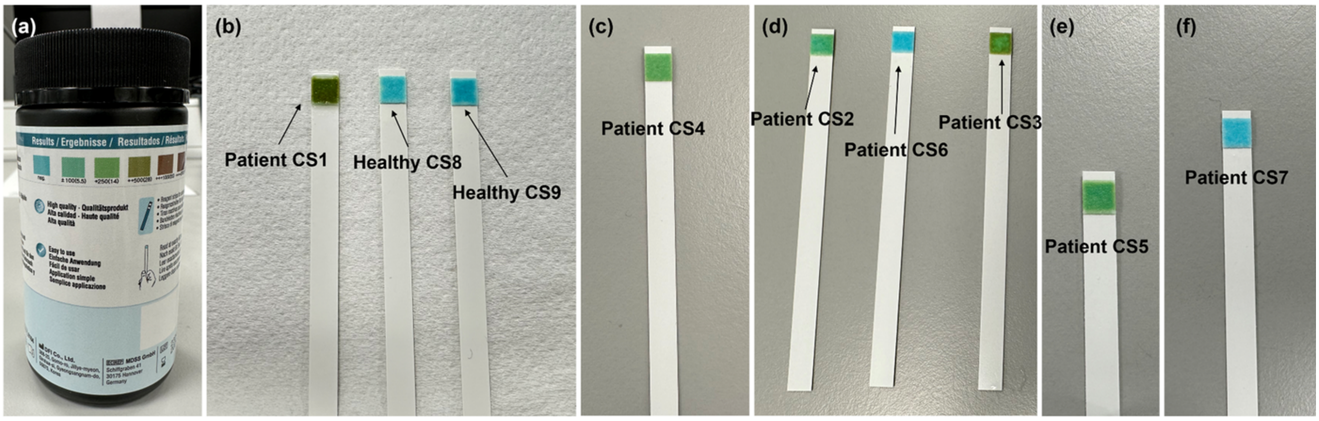

Figure S12. Test strips for detection of glucose in urine. (a) the colorimetric reference card on the vial label under good light. (b) Test strips, left: CKD patient; middle and right: healthy individuals. (c-f) Test strips from CKD patients.

POC analysis of urinary pH and glucose in clinical samples

Table S2. Determination of pH in human urine samples (n=3).

Sample	Found[i]	Detected	Difference[ii]	RSD (%)
CS1	5.38	5.57	+0.19	2.03
CS2	6.50	6.73	+0.23	2.43
CS3	5.00	5.15	+0.15	0.51
CS4	7.77	7.41	-0.36	4.17
CS5	7.00	6.67	-0.33	2.94
CS6	5.37	5.67	+0.30	2.28
CS7	5.25	5.38	+0.13	2.28
CS8	7.60	7.07	-0.53	3.30
CS9	5.23	4.83	-0.40	1.44

[i] The pH value of human urine samples tested by UK nationally accredited hospital chemical pathology laboratory and commercial pH meter.

[ii] Difference value = Detected value – Found value.

Table S3. Determination of glucose in human urine samples (n=3).

Sample[iii]	Found (μM)[iv]	Spiked (μM)	Detected (μM)	Recovery (%)[v]	RSD (%)
CS1	555.07	0	559.29	100.76	3.11
CS2	111.01	0	77.10	69.45	3.65
CS3	277.54	0	378.71	136.45	3.25
CS4	277.54	0	274.96	99.07	2.69
CS5	277.54	0	262.44	94.56	4.46
CS6	0	150	148.54	99.03	4.20
		300	316.95	105.65	5.37
		450	456.19	101.38	4.40
CS7	0	150	158.39	105.59	2.50
		300	294.27	98.09	1.67
		450	457.10	101.58	0.82
CS8	0	150	147.21	98.14	1.64
		300	306.70	102.23	1.21
		450	465.29	103.40	3.12
CS9	0	150	140.58	93.72	3.70
		300	320.38	106.79	5.11
		450	519.21	115.38	4.86

[iii] Sample CS1-CS7 is 50 times diluted CKD patient's urine sample. Samples CS8 and CS9 are urine samples from healthy individuals.

[iv] The value of glucose concentration of urine tested by dipstick methods.

[v] The ratio of detected value/(found value + spiked value).

References

[1] B. Conway and J. Mozota, "Surface and bulk processes at oxidized iridium electrodes—II. Conductivity-switched behaviour of thick oxide films," *Electrochimica Acta,* vol. 28, no. 1, pp. 9-16, 1983.

[2] C. Yang, C.-H. Wang, J.-S. Wu, and X. Xia, "Mechanism investigation of Prussian blue electrochemically deposited from a solution containing single component of ferricyanide," *Electrochimica acta,* vol. 51, no. 19, pp. 4019-4023, 2006.

[3] A. A. Karyakin, "Prussian blue and its analogues: electrochemistry and analytical applications," *Electroanalysis: An International Journal Devoted to Fundamental and Practical Aspects of Electroanalysis,* vol. 13, no. 10, pp. 813-819, 2001.

[4] A. Moya, R. Pol, A. Martínez-Cuadrado, R. Villa, G. Gabriel, and M. Baeza, "Stable full-inkjet-printed solid-state Ag/AgCl reference electrode," *Analytical chemistry,* vol. 91, no. 24, pp. 15539-15546, 2019.

[5] N. Rohaizad, C. C. Mayorga-Martinez, F. Novotný, R. D. Webster, and M. Pumera, "3D-printed Ag/AgCl pseudo-reference electrodes," *Electrochemistry Communications,* vol. 103, pp. 104-108, 2019.

[6] H. Lee, Y. J. Hong, S. Baik, T. Hyeon, and D. H. Kim, "Enzyme-based glucose sensor: from invasive to wearable device," *Advanced healthcare materials,* vol. 7, no. 8, p. 1701150, 2018.
