# Supplementary material for: A paper-based dual functional biosensor for safe and user-friendly point-of-care urine analysis
Source: Lab Chip. 2024 Apr 12;24(9):2454–67. doi: 10.1039/d4lc00163j (PMC11060138; doi:10.1039/d4lc00163j)

Electronic Supplementary Material (ESI) for Lab on a Chip.
This journal is © The Royal Society of Chemistry 2024

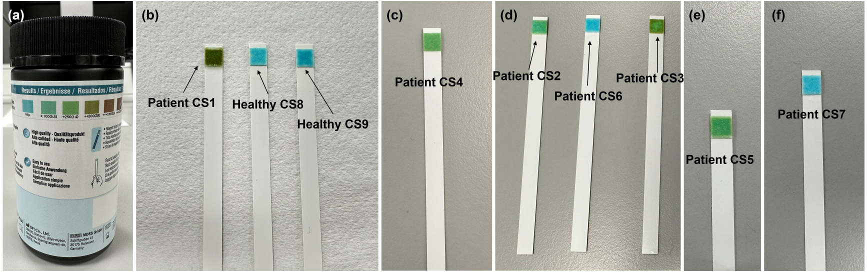

Supplement: LC-024-D4LC00163J-s010 [file LC-024-D4LC00163J-s010.pdf]
